# MEF2C protects bone marrow B-lymphoid progenitors during stress haematopoiesis

**DOI:** 10.1038/ncomms12376

**Published:** 2016-08-10

**Authors:** Wenyuan Wang, Tonis Org, Amélie Montel-Hagen, Peter D. Pioli, Dan Duan, Edo Israely, Daniel Malkin, Trent Su, Johanna Flach, Siavash K. Kurdistani, Robert H. Schiestl, Hanna K. A. Mikkola

**Affiliations:** 1 Department of Molecular, Cell and Developmental Biology, University of California Los Angeles, Los Angeles, California 90095, USA; 2 Eli and Edythe Broad Center for Regenerative Medicine and Stem Cell Research, UCLA, Los Angeles, California 90095, USA; 3 Molecular Biology Institute, UCLA, Los Angeles, California 90095, USA; 4 Institute of Molecular and Cell Biology, University of Tartu, Tartu 51010, Estonia; 5 Department of Molecular Toxicology, UCLA, Los Angeles, California 90095, USA; 6 Jonsson Comprehensive Cancer Center, UCLA, Los Angeles, California 90095, USA; 7 Department of Biological Chemistry, David Geffen School of Medicine at UCLA, Los Angeles, California 90095, USA; 8 The Eli and Edythe Broad Center for Regenerative Medicine and Stem Cell Research, University of California San Francisco, San Francisco, California 94143, USA

## Abstract

DNA double strand break (DSB) repair is critical for generation of B-cell receptors, which are pre-requisite for B-cell progenitor survival. However, the transcription factors that promote DSB repair in B cells are not known. Here we show that MEF2C enhances the expression of DNA repair and recombination factors in B-cell progenitors, promoting DSB repair, V(D)J recombination and cell survival. Although *Mef2c*-deficient mice maintain relatively intact peripheral B-lymphoid cellularity during homeostasis, they exhibit poor B-lymphoid recovery after sub-lethal irradiation and 5-fluorouracil injection. MEF2C binds active regulatory regions with high-chromatin accessibility in DNA repair and V(D)J genes in both mouse B-cell progenitors and human B lymphoblasts. Loss of *Mef2c* in pre-B cells reduces chromatin accessibility in multiple regulatory regions of the MEF2C-activated genes. MEF2C therefore protects B lymphopoiesis during stress by ensuring proper expression of genes that encode DNA repair and B-cell factors.

## Abstract (web summary)

MEF2C is a transcription factor required for B-cell proliferation. Here the authors show that MEF2C is also needed in B-cell development and recovery from stress by inducing expression of DNA repair factors that prevent double stranded breaks and enable VDJ recombination.

## Graphical abstract

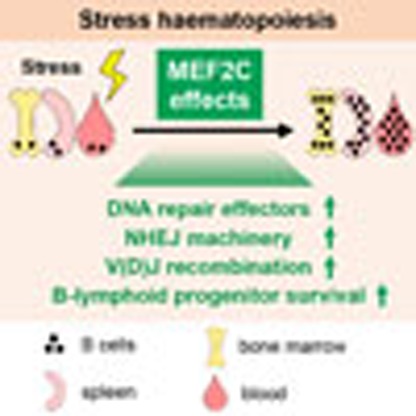

Sustained B lymphopoiesis through various stress conditions is essential for maintaining a functional immune system. B lymphopoiesis occurs in bone marrow where B-lymphoid progenitors undergo V(D)J recombination to generate B-cell receptors (BCRs)^1^,^2^,^3^,^4^. The success of V(D)J recombination is critical for humoral immunity as diverse BCRs are required to recognize antigens and generate antibodies. V(D)J recombination is initiated by creating DNA double strand breaks (DSBs) by RAG recombinases at the border of recombining gene segments^5^,^6^. After rearrangement, the DSBs are repaired by non-homologous end joining (NHEJ) machinery^7^,^8^. Defective DNA repair during this process results in cell death or genetic lesions^9^, making B lymphopoiesis inherently vulnerable. To ensure genomic integrity, B-lymphoid progenitors tightly regulate cell survival and exclude cells with abnormal rearrangement^10^. This homeostatic balance is altered during physiological ageing^11^,^12^,^13^ due to reduced V(D)J recombination efficiency^14^,^15^ and increased B-lymphoid progenitor death^16^, which contributes to the impaired immune function during ageing.

The haematopoietic system encounters various stress factors that necessitate rapid proliferation of stem/progenitor cells to replenish the blood/immune system^17^. The regeneration of the haematopoietic system under such situations is called stress haematopoiesis and can be induced by bone marrow transplantation^18^, radiation and chemotherapy^19^, bleeding^20^ and infection^21^. In addition to investigating the effects of stress on haematopoietic stem cell maintenance, several studies have focused on stress erythropoiesis and identified multiple unique signals that regulate this process^22^. However, little is known how other haematopoietic lineages secure proficient progenitor proliferation and differentiation during stress.

Studies have identified myocyte enhancer factor 2C (MEF2C) as a regulator of the B-lymphoid system. MEF2C is a MADS box transcription factor originally discovered as a regulator of cardiogenesis and myogenesis^23^. In bone marrow, *Mef2c* is highly expressed by common lymphoid progenitors (CLPs) and B-lymphoid cells, whereas *Mef2c* expression is minimal in T cells, granulocytes and erythrocytes^24^. Deletion of *Mef2c* by B-cell-specific Cd19-Cre showed that MEF2C is required for BCR-induced proliferation of splenic B cells^25^,^26^,^27^; however, as the deletion of *Mef2c* was not complete in bone marrow B-cell progenitors, this model cannot be used to evaluate the presence of B-cell progenitor defects. Deletion of *Mef2c* using Mx1-Cre and PIPC treatment followed by transplantation or culture led to a severe reduction in the number of B cells, whereas myeloid cell numbers were increased, indicating a role for MEF2C in myeloid/lymphoid fate choice^24^. We previously showed that haematopoietic deletion of *Mef2c* using Vav-Cre results in a reduction of bone marrow B-cell progenitors, especially pre-B cells, without overtly affecting the peripheral B-cell pool during homeostasis^28^. A requirement for MEF2C within bone marrow B-lymphoid cells was also documented using B-cell-specific Mb-1-Cre. This led to a reduction of B cells in both bone marrow and spleen of neonates. Although peripheral cellularity of B cells was corrected in adult mice, bone marrow B lymphopoiesis remained compromised^29^. Another study showed that MEF2C acts redundantly with MEF2D, and that MEF2C/D are activated by pre-BCR signalling. Chromatin immunoprecipitation-sequencing (ChIP-seq) analysis showed that MEF2C directly binds to several pre-B-cell genes, and possibly regulates them together with other B-cell regulators such as E2A and IKAROS^30^. Although these studies show a requirement for MEF2C in B-lymphoid progenitors, the cellular and molecular mechanisms through which MEF2C protects bone marrow B lymphopoiesis are mostly unknown. Moreover, little is known about MEF2C function during stress. This question is particularly intriguing as *Mef2c*-deficient mice share features of B-lymphoid ageing^28^, which is characterized by reduction of bone marrow B-cell progenitors, whereas peripheral B-cell cellularity can initially be maintained through compensatory mechanisms^11^,^12^,^13^.

Here we report that MEF2C protects bone marrow B-lymphoid progenitors by augmenting the transcription of factors crucial for DNA repair and V(D)J recombination, thereby ensuring efficient bone marrow B lymphopoiesis. Loss of *Mef2c* severely compromises B-lymphoid recovery after sub-lethal irradiation or 5-fluorouracil (5-FU) injection, documenting a critical function for MEF2C during regenerative stress. We find that MEF2C binds directly to regulatory regions of genes encoding DNA repair and V(D)J factors, as well as B-cell transcription factors in mouse B-cell progenitors and human B-lymphoblasts, co-localizing with epigenetic marks representing active enhancers and promoters. ATAC-sequencing (ATAC-seq) shows that MEF2C is required for proper chromatin accessibility of regulatory regions of its target genes in mouse pre-B cells. These findings uncover a central role for MEF2C as a transcriptional activator of DNA repair and B-cell regulatory machinery in B-lymphoid progenitors, and establish that MEF2C-dependent transcriptional mechanisms are required to secure efficient bone marrow B-cell production during stress haematopoiesis.

## Results

### MEF2C maintains the cellularity of B-lymphoid progenitors

**Figure 1:**
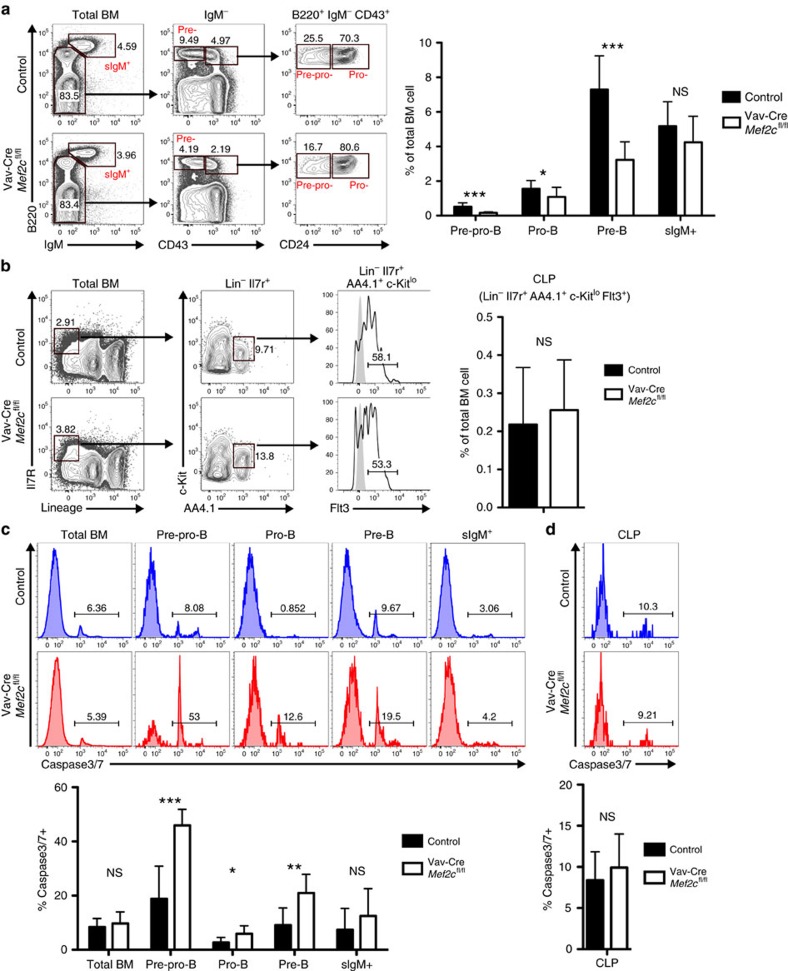
MEF2C maintains the integrity of bone marrow B-lymphoid compartment. (**a**) Flow cytometric analysis of bone marrow B-lymphoid progenitor fractions in Vav-Cre *Mef2c*^fl/fl^ mice as compared with control mice revealed reduction of all B-lymphoid progenitors in *Mef2c*-deficient mice while the mature B cells in the bone marrow (sIgM^+^) were not affected (*n*≥11). (**b**) Flow cytometric analysis suggested that loss of *Mef2c* does not affect the frequency of bone marrow common lymphoid progenitors (CLP) (*n*≥7). (**c**) Flow cytometric analysis of caspase 3/7 activation in bone marrow B-lymphoid progenitors documented increased cell death in *Mef2c*-deficient B-lymphoid progenitors while total bone marrow and sIgM^+^ cells were not affected (*n*≥7). (**d**) Caspase 3/7 activation was not increased in *Mef2c*-deficient CLP (*n*=8). All mice were analysed at 7–11 months of age and both male and female mice were included. Data shown are the mean±s.d. of two or more independent experiments. NS, not significant, *P<0.05, **P<0.01 and ***P<0.001, unpaired *t*-test.

To define the processes that MEF2C regulates in B-lymphoid cells, *Mef2c*^fl/fl^ was conditionally deleted in mice using Vav-Cre^28^,^31^. Unless otherwise stated, middle-aged mice (7–11-month-old, males and females) were used for the analyses, as our prior study showed that the reduction in *Mef2c*-deficient B-cell progenitors was most notable at this age^28^. Analysis of larger groups of mice confirmed a reduction of bone marrow B cells in *Mef2c*-deficient mice, while B-lymphocytes in blood and spleen were not significantly affected (Supplementary Fig. 1). In addition to the reduction of pre-pro-B and pre-B cells in *Mef2c*-deficient mice observed previously^28^, analysis of a larger cohort of mice suggested that all bone marrow B-lymphoid progenitor stages (pre-pro-B, pro-B and pre-B) were significantly reduced (Fig. 1a). The reduction of all bone marrow B-cell progenitor subsets was also confirmed by immunophenotyping B-cell development as defined by Hardy *et al.*^32^ (Supplementary Fig. 2a). In contrast, the cellularity of mature recirculating B cells (sIgM^+^ or Fr.F) in bone marrow was not affected (Fig. 1a). Moreover, Vav-Cre *Mef2c*^fl/fl^ mice showed no significant difference in the percentage of CLPs, defined as Lin^−^c-Kit^lo^AA4.1^+^IL-7Rα^+^Flt3^+^ (Fig. 1b) or Lin^−^IL-7R^+^Sca-1^lo^c-Kit^lo^ (Supplementary Fig. 2b). These data suggested that MEF2C has a critical function in maintaining the integrity of bone marrow B-lymphoid progenitor compartment.

Analysis of bone marrow B-cell progenitors at different ages showed a reduction already in young and middle-aged *Mef2c*-deficient mice. By 8–10 months of age, *Mef2c*-deficient mice exhibited a comparable decrease in pre-B-cell percentages as controls showed by 20–22 months (Supplementary Fig. 3). These results raised the question if MEF2C controls similar molecular and cellular processes that are negatively affected during B-cell ageing.

Regulation of cell viability is a critical mechanism by which B-lymphoid progenitors maintain quality control^10^. Analysis of caspase 3/7 activation revealed increased cell death of all bone marrow B-lymphoid progenitor subsets in *Mef2c*-deficient mice. This effect was specific to the B-cell progenitor compartment as sIgM^+^ mature B-lymphocytes, total bone marrow cells and CLPs were unaffected (Fig. 1c,d). These data suggest that MEF2C protects bone marrow B-lymphoid progenitor survival. *Bcl2l1*(Bcl-x_L_) is a known pro-survival factor for bone marrow B-cell progenitors and has previously been shown to be regulated by MEF2C in mature B cells^26^,^33^. Quantitative reverse transcription–PCR (Q-PCR) revealed that MEF2C is required for normal expression of *Bcl2l1* in both pro- and pre-B cells (Supplementary Fig. 2c), further validating the importance for MEF2C in regulating B-cell progenitor survival.

### MEF2C is essential for B-lymphoid recovery during stress

**Figure 2:**
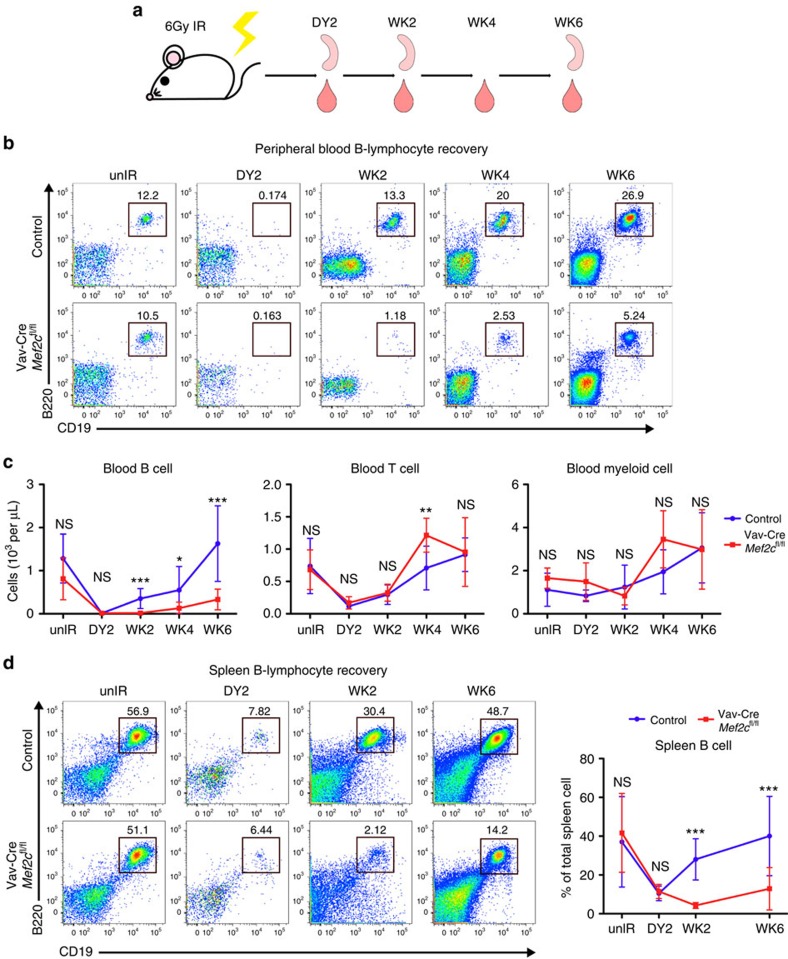
MEF2C supports efficient peripheral B-cell recovery on sub-lethal irradiation. (**a**) Experimental design of haematopoietic ablation by sub-lethal irradiation: Vav-Cre *Mef2c*^fl/fl^ and control mice received 6 Gy of total body irradiation. Peripheral blood was collected for complete blood cytometry (CBC) and flow cytometric analysis at day 2 and weeks 2, 4 and 6. Mice were killed and spleens were analysed by flow cytometry at day 2, and weeks 2 and 6. (**b**) Representative flow cytometric analysis of peripheral blood B cells revealed defective B-cell recovery in *Mef2c*-deficient mice. (**c**) Quantification of peripheral blood lineage cell count (WBC count from CBC was multiplied with each lineage-specific percentage obtained from flow cytometry) documents that loss of *Mef2c* compromised the recovery of B cells while T and myeloid cells were not affected. (**d**) Flow cytometric analysis of spleen B cells in both Vav-Cre *Mef2c*^fl/fl^ and control mice after irradiation revealed defective B-cell recovery in *Mef2c*-deficient spleens. All mice were analysed at 7–10 months of age and both male and female mice were included. Day 2: *n*=4 mice, data shown are the mean±s.d. of two independent experiments; other time points: *n*≥5, data shown are the mean±s.d. of three or more independent experiments. NS, not significant, *P<0.05, **P<0.01 and ***P<0.001, unpaired *t*-test.

Since loss of *Mef2c* compromised the survival and cellularity of bone marrow B-lymphoid progenitors during homeostasis, we asked if these defects are exacerbated during stress. As sub-lethal irradiation ablates most haematopoietic cells and leads to rapid proliferation and differentiation of stem/progenitor cells, MEF2C requirement in haematopoietic recovery was first assessed following 6Gy sub-lethal irradiation (Fig. 2a). Two days after irradiation, loss of blood B, T and myeloid cells was observed in both *Mef2c*-deficient and control mice (Fig. 2b,c, Supplementary Fig. 4a,b). No significant difference in total white blood cell (WBC) or red blood cell counts was observed in *Mef2c*-deficient mice compared with controls before or after irradiation. The previously identified reduction in platelets^28^ was retained after irradiation (Supplementary Fig. 4c). However, while control mice regained normal levels of B cells within 6 weeks after irradiation, *Mef2c*-deficient mice showed minimal B-lymphoid recovery at this stage (Fig. 2b,c). In contrast, the recovery of T-lymphoid and myeloid lineages in *Mef2c*-deficient mice was comparable to controls (Fig. 2c, Supplementary Fig. 4a,b). The spleens of *Mef2c*-deficient animals also demonstrated impaired B-lymphoid recovery, excluding the sequestration of B cells in peripheral lymphoid organs as a major cause to the impaired recovery of circulating B cells (Fig. 2d).

**Figure 3:**
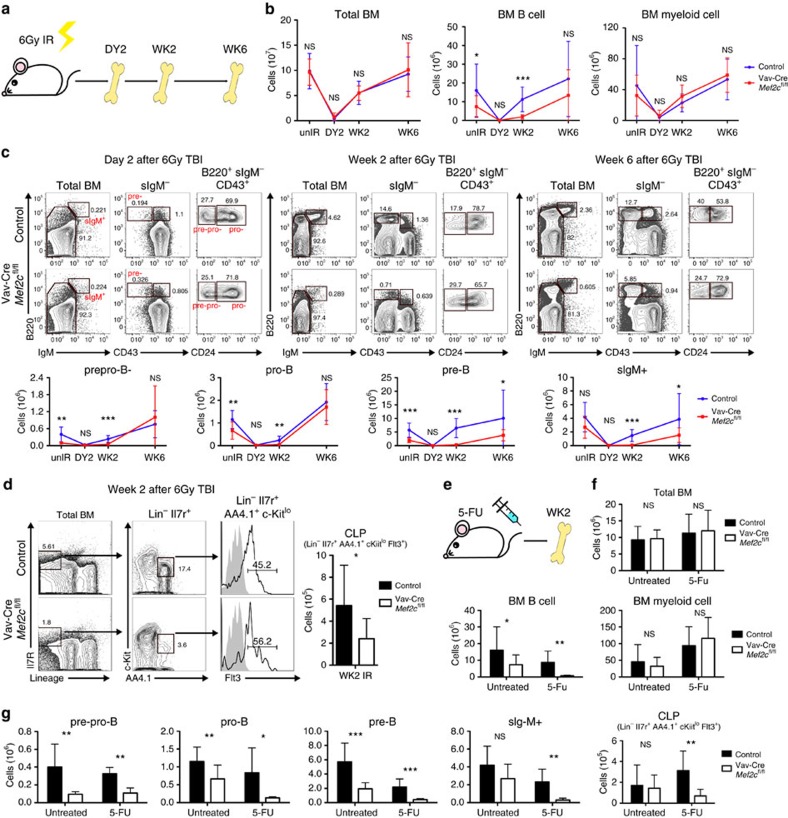
Pre-B-cell stage is a bottleneck for B-cell recovery in *Mef2c*-deficient bone marrow during stress. (**a**) Experimental design of bone marrow recovery analysis: Vav-Cre *Mef2c*^fl/fl^ and control mice received 6 Gy of total body irradiation. Mice were killed and bone marrow compartments were analysed by flow cytometry at day 2 and weeks 2 and 6. (**b**) Quantification of total bone marrow, bone marrow B and myeloid cellularity shows comparable total bone marrow and myeloid cell counts in *Mef2c-*deficient and control mice while reduced bone marrow B-cell counts in *Mef2c-*deficient mice during recovery from irradiation. (**c**) Representative flow cytometric analysis and quantification of bone marrow B-lymphoid progenitors at 2 days, 2 weeks and 6 weeks post 6 Gy irradiation shows that loss of *Mef2c* compromises most drastically the recovery of pre-B cells and downstream sIgM^+^ bone marrow B cells. (**d**) Quantification of CLPs at 2 weeks post 6 Gy irradiation shows that *Mef2c* is required for proper CLP recovery on IR-induced stress. (**e**) Experimental design of 5-FU stress analysis: Vav-Cre *Mef2c*^fl/fl^ and control mice received sub-lethal dose of 5-FU and bone marrow was collected at week 2 for flow cytometry. (**f**) Quantification of total bone marrow and B and myeloid cellularity shows comparable total bone marrow and myeloid cell counts in *Mef2c-*deficient and control mice, whereas bone marrow B-cell counts were reduced in *Mef2c-*deficient mice during recovery from 5-FU. (**g**) Quantification of bone marrow B-cell progenitors and CLPs at week 2 post 5-FU injection revealed compromised recovery of all B-cell progenitors and CLPs in *Mef2c-*deficient mice. All mice were analysed at 5–10 months of age, and both male and female mice were included. Day 2: *n*=4, data shown are the mean±s.d. of two independent experiments; other time points: *n*≥5, data shown are the mean±s.d. of three or more independent experiments. NS, not significant, *P<0.05, **P<0.01 and ***P<0.001, unpaired *t*-test.

To understand the compromised peripheral B-lymphoid recovery in *Mef2c*-deficient mice during irradiation-induced stress, the kinetics of bone marrow recovery was assessed (Fig. 3a). At 2 days after irradiation, the cellularity of bone marrow B-lymphoid progenitors was greatly reduced in both *Mef2c*-deficient and control mice. Although the recovery of total bone marrow BM cells was comparable between *Mef2c-*deficient mice and controls, the recovery of bone marrow B cells was markedly impaired in the absence of *Mef2c* (Fig. 3b, Supplementary Fig. 5a,b). By 2 weeks after irradiation, the cellularity of all bone marrow B-lymphoid progenitors in controls had drastically improved, and by 6 weeks reached a level comparable to unirradiated mice (Fig. 3c). In contrast, there was minimal recovery of all B-cell progenitor subsets in *Mef2c*-deficient mice at week 2. *Mef2c* loss also compromised CLP recovery 2 weeks post irradiation (Fig. 3d). By week 6, levels of pre-pro-B and pro-B cells in irradiated *Mef2c-*deficient mice were similar to unirradiated mice. This was not the case for pre-B cells and sIgM^+^ B cells that were still severely reduced (Fig. 3c). These data suggested that the impaired recovery of peripheral B cells in *Mef2c*-deficient mice during irradiation-induced stress haematopoiesis results from inefficient bone marrow B lymphopoiesis that is affected already at the level of CLP, and pinpointed the pre-B-cell stage as a major bottleneck in B-lymphoid recovery.

To investigate whether the poor regeneration of B-lymphoid cells in *Mef2c*-deficient mice correlates with increased cell death, viability of bone marrow B-cell progenitors was assessed. Caspase 3/7 staining 2 weeks after irradiation showed increased cell death in *Mef2c*-deficient B-lymphoid progenitors (Supplementary Fig. 5c), documenting a critical requirement for MEF2C in protecting bone marrow B-lymphoid progenitor survival on regenerative stress.

To test if MEF2C governs stress haematopoiesis caused by other factors, bone marrow recovery of control and *Mef2c*-deficient mice was analysed after 5-FU injection, which ablates cycling haematopoietic cells and promotes rapid proliferation and differentiation of remaining haematopoietic cells (Fig. 3e). Similar to the recovery from irradiation, the recovery of bone marrow B cells, but not total bone marrow or bone marrow myeloid cells, was compromised in *Mef2c*-deficient mice at week 2 (Fig. 3f). Analysis of bone marrow progenitor subsets revealed that MEF2C is required for proper B-lymphoid progenitor and CLP recovery on 5-FU induced stress haematopoiesis (Fig. 3g). These data collectively suggest that MEF2C is required to protect B-lymphoid progenitors during regenerative stress, even in the absence of DNA damage caused by external insults such as irradiation.

### MEF2C regulates DNA DSB repair in B-lymphoid progenitors

To determine the molecular basis for the inefficient B lymphopoiesis in *Mef2c*-deficient bone marrow, gene expression microarray analysis was used to identify the pathways regulated by MEF2C. The analysis was first focused on pre-B cells, which showed most drastic defects in the recovery from irradiation in *Mef2c*-deficient bone marrow.

**Figure 4:**
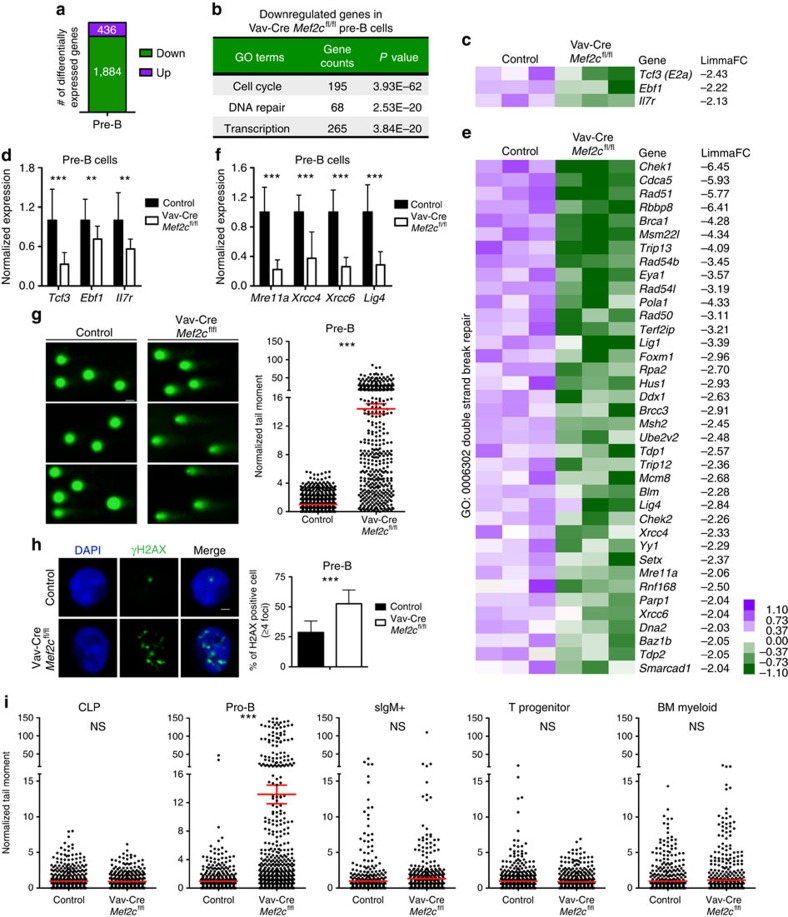
MEF2C regulates DNA double strand break repair in B-cell progenitors. (**a**) Microarray analysis of pre-B cells from control and Vav-Cre *Mef2c*^fl/fl^ mice (9-months old) revealed 1,884 significantly (|FC|≥2 and *P*≤0.05) downregulated and 436 upregulated genes in the absence of *Mef2c* (*n*=3). (**b**) Cell cycle, DNA repair and transcription were among the most significantly enriched GO categories of genes downregulated in *Mef2c*-deficient pre-B cells. (**c**,**d**) Microarray and Q-PCR analysis of selected B-cell regulator that are significantly downregulated in *Mef2c*-deficient pre-B cells. (**e**) Microarray analysis of DNA DSB repair genes that are significantly downregulated in *Mef2c*-deficient pre-B cells are shown. (**f**) Q-PCR of key genes encoding DNA repair machinery validated defective expression in *Mef2c-*deficient pre-B cells. (**d**,**f**) *n*≥5 mice, data shown are the mean±s.d. of two or more independent experiments. **P<0.01, ***P<0.001, unpaired *t*-test. (**g**) Representative figures and quantification of alkaline comets revealed increased DNA damage in *Mef2c*-deficient pre-B cells (7–10-month old). *n*≥6 mice, data shown are the mean±s.e.m. of three independent experiments. ***P<0.001, unpaired *t*-test. About 728 control pre-B cells and 450 *Mef2c*-deficient pre-B cells were analysed. Scale bar, 20 μm. (**h**) Representative IF analysis of γH2AX in pre-B cells (7–10-months old) revealed that MEF2C is required for proper DSB repair in pre-B cells. *n*≥6 mice, data shown are the mean±s.d. of three independent experiments. Scale bar, 1 μm. ***P<0.001, unpaired *t*-test. (**i**) Quantification of alkaline comets revealed increased DNA damage in pro-B cells, but not CLP, sIgM^+^ bone marrow B cells, thymic T progenitors (DN1 and 2) or bone marrow myeloid cells in *Mef2c*-deficient mice (7–10-months old). *n*≥5 mice and data shown are the mean±s.e.m. of two or more independent experiments. More than 350 cells for each population were analysed. NS, not significant, **P<0.01, ***P<0.001, unpaired *t*-test. Both male and female mice were included for all experiments.

Lack of *Mef2c* significantly (|FC|≥2.0, *P* value≤0.05) reduced the expression of 1,884 genes and increased the expression of 436 genes in pre-B cells (Fig. 4a, Supplementary Data 1). Gene ontology (GO) analysis of differentially expressed genes identified cell cycle, DNA repair and transcription processes among the most significantly enriched categories of the genes downregulated in *Mef2c*-deficient pre-B cells (Fig. 4b); few significant GO categories were identified among the upregulated genes (Supplementary Data 1). These data suggest that MEF2C primarily functions as an activator in bone marrow B-cell progenitors.

The genes that were downregulated in *Mef2c-*deficient pre-B cells included critical B-cell regulators: *Il7r,* which was previously identified as a direct target of MEF2C^24^, as well as *Tcf3*(*E2a*) and *Ebf1*, transcriptional regulators of B-lymphoid progenitor differentiation and V(D)J recombination^34^,^35^,^36^,^37^,^38^,^39^,^40^ (Fig. 4c). Their downregulation was also verified by Q-PCR (Fig. 4d).

As B-lymphoid progenitor development, survival and V(D)J recombination also require DNA DSB repair factors, the expression of DSB repair genes was examined. Analysis of microarray data suggested that loss of *Mef2c* affects both NHEJ and homologous recombination (HR) repair pathways. As such, the transcription of DSB sensors (*Mre11a* & *Rad50*) and effectors of both NHEJ (*Xrcc4*, *Xrcc6* and *Lig4*) and HR (*Chek1*&*2*, *Rad51* and *Rad54l*) was significantly downregulated in *Mef2c*-deficient cells (Fig. 4e). Reduced expression of selected DSB repair genes (*Mre11a*, *Xrcc4*, *Xrcc6* and *Lig4*) was validated by Q-PCR in pre-B cells (Fig. 4f).

To assess the functional requirement for MEF2C in DNA DSB repair, alkaline comet assay, which detects DNA strand breaks, was performed. Qualitative and quantitative analyses of comet tails revealed increased levels of DNA damage in *Mef2c*-deficient pre-B cells (Fig. 4g). To assay DSB repair specifically, immunostaining for γH2AX was conducted. Quantification of γH2AX foci revealed increased DNA DSBs in *Mef2c*-deficient pre-B cells (Fig. 4h), confirming the requirement for MEF2C in proper DSB repair in bone marrow B-lymphoid progenitors.

Although pre-B-cell stage was a major bottleneck in *Mef2c-*deficient B-lymphoid recovery during stress, the survival of other B-cell progenitors was also reduced in *Mef2c*-deficient mice. We thus asked if MEF2C regulates DNA repair in other B-cell progenitors. Microarray and Q-PCR analysis of pro-B cells suggested that MEF2C also transcriptionally regulates DNA repair machinery in these cells (Supplementary Fig. 6, Supplementary Data 2).

To further define the stages of haemato-lymphoid development in which MEF2C functions to enhance DNA repair, alkaline comet assay was performed on various haematopoietic subpopulations. Similar to pre-B cells, lack of *Mef2c* led to increased DNA damage in pro-B cells, whereas the upstream CLPs or downstream bone marrow sIgM^+^ mature B cells were not affected (Fig. 4i). Moreover, lack of *Mef2c* did not affect the level of DNA damage in thymic T-cell progenitors or bone marrow myeloid cells (Fig. 4i). These data documented that MEF2C is required for efficient DNA repair in bone marrow B-lymphoid progenitors.

### MEF2C ensures efficient BCR rearrangement

**Figure 5:**
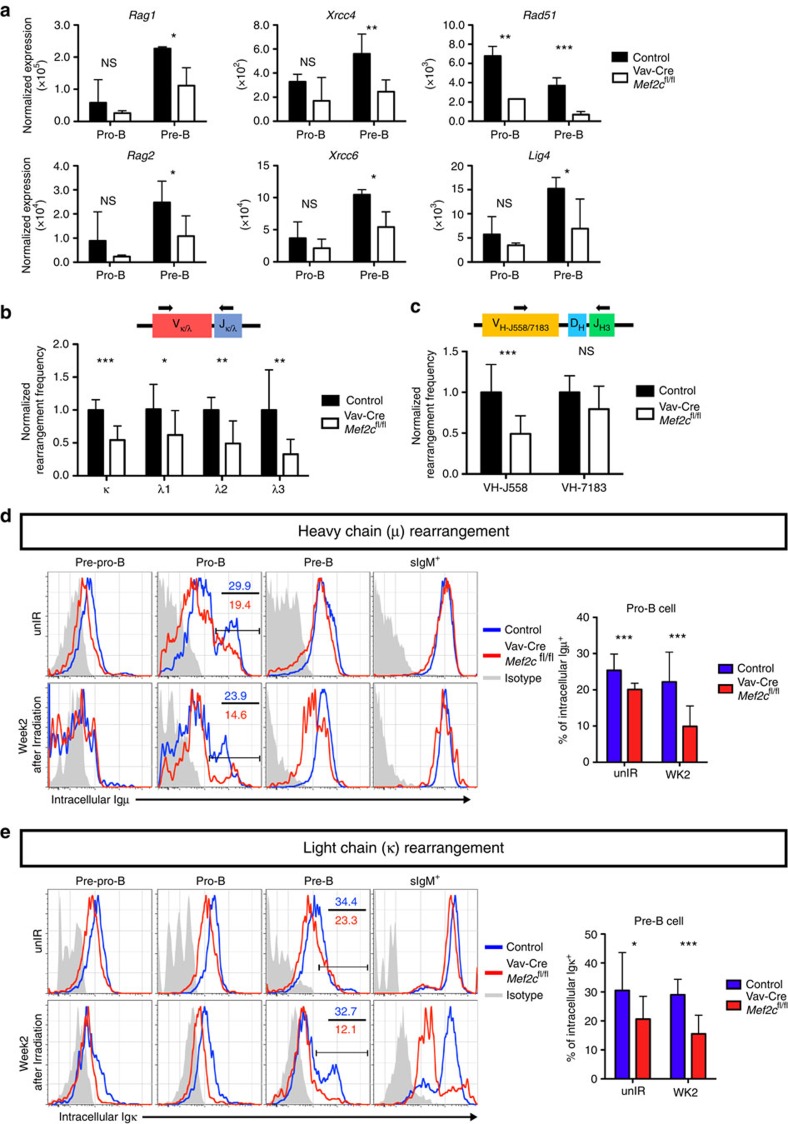
MEF2C promotes BCR rearrangement during bone marrow B lymphopoiesis. (**a**) Analysis of Affymetrix microarray data shows the expression of *Rag1*, *Rag2*, *Xrcc4, Xrcc6*, *Lig4* and *Rad51* in pro-B and pre-B cells in both control and Vav-Cre *Mef2c*^fl/fl^ mice (9-months old). Loss of *Mef2c* compromised the expression of all these factors (*n*≥2). (**b**,**c**) Quantitative PCR analysis of κ and λ light chain rearrangement (*n*≥5 mice) in bone marrow pre-B cells, and V_H_-J558 and V_H_-7183 to DJ_H3_ heavy chain rearrangement (*n*≥7) in pro-B cells (7–10-months old) revealed significantly reduced frequencies of κ and λ light chain and V_H_-J558 heavy chain recombination in the absence of *Mef2c*. (**d**,**e**) Representative flow cytometric plots and quantification of intracellular expression of μ and κ in bone marrow B-cell progenitors from mice without irradiation and 2 weeks after 6 Gy total body irradiation shows compromised rearrangement efficiency already in steady state, and further exaggeration of the defect during stress haematopoiesis (7–10-months old, *n*≥10). Both male and female mice were included. Data shown are the mean±s.d. of three or more independent experiments. *P<0.05, **P<0.01 and ***P<0.001, unpaired *t*-test.

In addition to maintaining genomic integrity, DNA repair is also required for V(D)J recombination during B-lymphoid differentiation. The success of V(D)J recombination is critical for B-cell progenitor survival, and requires proper expression of recombination factors^37^. A previous study suggested that *Mef2c* loss impairs the induction of *Rag1* in bone marrow multipotent progenitors^24^. Analysis of *Mef2c*-deficient pro-B and pre-B cells showed reduced expression of *Rag1*, *Rag2* and genes encoding NHEJ machinery (*Xrcc4*&*6* and *Lig4*) (Fig. 5a). These results suggest that MEF2C promotes V(D)J recombination by enabling efficient induction of effectors that execute this process.

To determine if the compromised expression of V(D)J recombination factors in *Mef2c*-deficient mice had any downstream effect on immunoglobulin rearrangement, we performed Q-PCR analysis to assess the rearrangement frequency of heavy and light chains. First we assessed recombination of κ and λ light chains, which occur at the pre-B-cell stage. Genomic DNA was extracted from FACS-sorted pre-B cells and subjected to SYBR green Q-PCR using primers that detect only the rearranged κ or λ alleles. The abundance of rearranged κ and λ chain was determined relative to the constant genomic DNA region of *Actb* and normalized to control mice. Loss of *Mef2c* significantly reduced the rearrangement efficiency of both κ and λ light chains (Fig. 5b).

While light chain rearrangement occurs in pre-B cells, heavy chain rearrangement occurs in pre-pro- and pro-B cells. Since *Mef2c*-deficient pro-B cells also showed defective DNA repair and downregulation of similar biological pathways as in pre-B cells, MEF2C function in heavy chain rearrangement was also tested. Q-PCR analysis in *Mef2c*-deficient pro-B cells revealed a significant reduction in V to DJ rearrangement frequency of the most frequent IgH family in adult mice (V_H_-J558)^41^ (Fig. 5c), while the heavy chain family V_H_-7183 demonstrated a non-significant trend towards being reduced. These data suggest that MEF2C is required for efficient heavy and light chain recombination in bone marrow B-lymphoid progenitors.

We next quantified the rearranged heavy (μ and light (κ) at protein level. As the execution of V(D)J recombination is also required for the rapid regeneration of B-lymphoid progenitors during regenerative stress, we performed the analysis both before and 2 weeks after sub-lethal irradiation. Control mice were able to efficiently execute heavy and light chain rearrangement both during homeostasis and regenerative stress (Fig. 5d,e). In contrast, the protein levels of intracellular μ and κ in *Mef2c*-deficient pro- and pre-B cells, respectively, were already reduced at steady state. These defects were more drastic when assessed 2 weeks after irradiation (Fig. 5d,e). As such, MEF2C is critical for efficient V(D)J recombination in bone marrow B-lymphoid progenitors during regenerative stress.

### MEF2C directly binds to DNA repair and V(D)J factor genes

To determine whether MEF2C binds to the regulatory elements of genes encoding DNA repair and V(D)J recombination factors, we first analysed ChIP-seq data for MEF2C in human B-lymphoblasts (Encode)^42^. GO analysis of MEF2C-bound genes in human B-lymphoblasts revealed significant enrichment of transcription regulation, cell cycle regulation and B-cell differentiation categories (Supplementary Fig. 7a). Intersection of the human ChIP-seq data with mouse microarray data revealed that a considerable fraction of genes downregulated in *Mef2c*-deficient mouse pre-B cells were associated with MEF2C peaks in human B-lymphoblasts. Moreover, this gene set was enriched for genes that function in transcription regulation and DNA repair (Supplementary Fig. 7a).

The majority of MEF2C-binding sites were distally located (5 to 500 kb) relative to transcription start sites (TSSs), while 8% of MEF2C binding localized to within 5 kb of TSSs (Supplementary Fig. 7b), indicating that MEF2C can function at enhancers and promoters. Analysis of human B-lymphoblast ChIP-seq data for histone modifications and the transcriptional co-activator p300 showed that MEF2C binding was strongly correlated with a ‘pro-transcription' chromatin state. MEF2C binding co-localized with co-activator p300 and histone marks representative of an ‘activating' transcriptional environment, including H3K4me1, H3K4me3 and H3K27ac. No enrichment of repressive histone marks H3K9me3 or H3K27me3 was observed around MEF2C-binding sites (Supplementary Fig. 7c). These data further suggest that MEF2C primarily acts as a transcriptional activator in B-lymphoid cells. Analysis of individual MEF2C candidate target genes revealed that MEF2C directly binds to genes encoding RAG recombinases, DSB sensor components (MRE11A and RAD50), NHEJ effectors (XRCC4 and LIG4), HR effectors (RAD51AP1) and B-cell regulator (IL7R, TCF3 and EBF1). MEF2C peaks were associated with p300 binding and activation-associated chromatin marks at these genes, suggesting that MEF2C directly promotes their transcription (Supplementary Fig. 7d).

**Figure 6:**
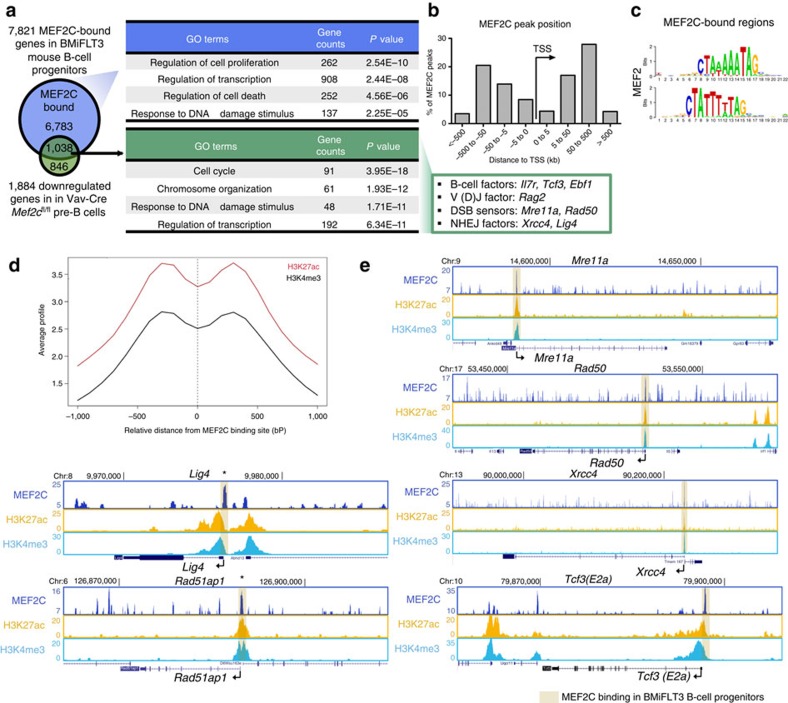
MEF2C binds to DNA repair and B-cell regulators in mouse B-cell progenitors. (**a**) Intersection of MEF2C-bound genes in mouse BMiFLT3 B-cell progenitors and downregulated genes in *Mef2c*-deficient mouse bone marrow pre-B cells. (**b**) MEF2C-binding sites are located both around TSS and at distant regulatory elements in mouse B-cell progenitors. (**c**) MEF2 motif is the third most enriched motif in MEF2C-binding sites in BMiFLT3 cells. (**d**) Genome browser tracks show MEF2C binding, H3K27ac and H3K4me3 enrichment at genes encoding DNA repair and B-cell regulators. MEF2C bindings are highlighted in brown. MEF2C bindings that do not yield statistical significance are labelled with asterisks. (**e**) MEF2C-binding sites in mouse B-cell progenitors correlate with active epigenetic marks.

To assess MEF2C binding in mouse B-cell progenitors, we also analysed a recently published ChIP-seq data set in BMiFLT3 B-cell progenitors^30^. Similar to human B-lymphoblasts, MEF2C binding in mouse B-cell progenitors was observed at genes that regulate transcription and DNA damage response (Fig. 6a). Moreover, the binding sites located both at promoters and distal regions (Fig. 6b), and were enriched for MEF2 motifs (Fig. 6c). More than half of the genes downregulated in *Mef2c-*deficient pre-B cells were bound by MEF2C in BMiFLT3 cells, and they were enriched in genes associated with response to DNA damage stimulus and cell cycle regulation (Fig. 6a). These candidate direct target genes included B-cell factors (*Il7r, Tcf3* and *Ebf1*), V(D)J initiator (*Rag2*), DSB sensors (*Mre11a* and *Rad50*) and NHEJ effectors (*Xrcc4* and *Lig4*). When intersecting MEF2C binding in BMiFLT3 cells with ChIP-seq data of active histone marks in mouse bone marrow pre-B cells, enrichment of active histone marks H3K27ac and H3K4me3 around MEF2C-binding sites was observed both in global analysis (Fig. 6d), and at individual MEF2C target genes (Fig. 6e.)

### *Mef2c* loss affects chromatin accessibility in pre-B cells

**Figure 7:**
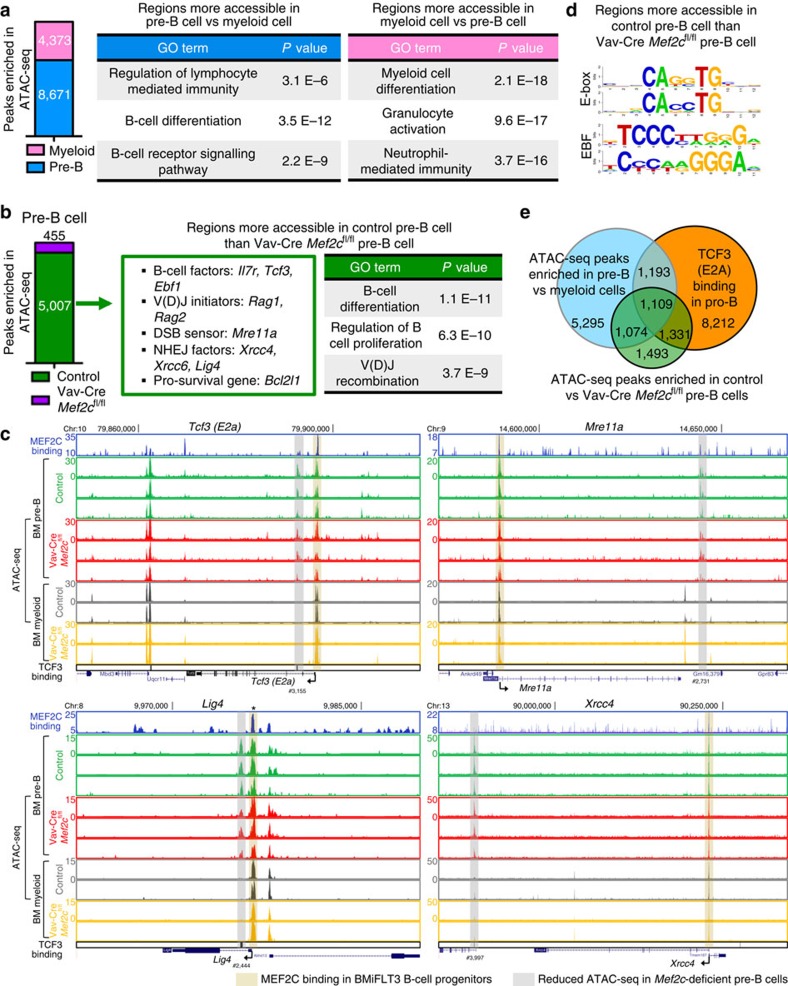
MEF2C promotes chromatin accessibility of its target genes in mouse pre-B cells. (**a**) MAnorm analysis of ATAC-sequencing(ATAC-seq) data in control pre-B cells and control bone marrow myeloid (Mac1^+^Gr1^+^) revealed that the accessibility of regulatory sites in lineage-specific genes is differentially (ratio≥4, *P* value≤0.01) regulated in these two populations. (**b**) MAnorm analysis of ATAC-seq data in control and *Mef2c-*deficient pre-B cells revealed that MEF2C is required to promote chromatin accessibility in regulatory regions of genes required for B-cell differentiation. (**c**) Loss of *Mef2c* reduced the chromatin accessibility nearby genes that are critical for B-cell differentiation and DNA repair. Many of these regulatory sites are also uniquely accessible in pre-B cells as compared with myeloid cells. Regions that are significantly (ratio≥4, *P* value≤0.01) more accessible in control than *Mef2c-*deficient pre-B cells are highlighted in grey and labelled with reference numbers co-related to Supplementary Data 4. Regions that are bound by MEF2C are highlighted in brown. MEF2C bindings that do not yield statistical significance are labelled with asterisks. (**d**) The ATAC-seq peaks that are more accessible in control pre-B cells compared with *Mef2c-*deficient pre-B cells are enriched for E-box and EBF motifs. (**e**) Venn diagram showing extensive overlap of regions that are more accessible in control pre-B cells vs. *Mef2c-*deficient pre-B cells with regions that are more accessible in pre-B cells than myeloid cells, as well as TCF3-bound regions in pro-B cells.

To investigate how MEF2C promotes gene expression, ATAC-seq analysis was performed to compare open chromatin regions in control and *Mef2c-*deficient bone marrow pre-B cells and myeloid cells. MAnorm^43^ analysis of regions that show difference in chromatin accessibility (ratio≥4, *P* value≤0.01) in pre-B cells and control myeloid cells revealed that both cell types possess lineage-specific regulatory regions that are more accessible (8671 in pre-B cells, and 4,372 in myeloid cells; Fig. 7a, Supplementary Data 3). GO analysis confirmed that regions more accessible in pre-B cells are enriched for B-cell differentiation pathways while regions more accessible in myeloid cells are enriched in myeloid differentiation pathways (Fig. 7a). These data reveal novel cell type-specific regulatory regions, and imply that regulation of chromatin accessibility plays a role in lineage differentiation.

To determine if MEF2C is involved in modulating chromatin accessibility of its target genes in B-cell progenitors, MAnorm analysis was performed in control versus *Mef2c-*deficient pre-B cells. In agreement with MEF2C being a transcriptional activator in B-cell progenitors, significantly higher number of regions showed increased accessibility in control (5,007) than *Mef2c-*deficient pre-B cells (455) (Fig. 7b, Supplementary Data 4). The sites that were less accessible in *Mef2c-*deficient pre-B cells include regions associated with MEF2C target genes that are important in B-cell differentiation (*Il7r, Tcf3* and *Ebf1*), V(D)J initiation (*Rag1*&*2*), DSB repair (*Mre11a, Xrcc4, Xrcc6* and *Lig4*) and B-cell progenitor survival (*Bcl2l1*). GO analysis of genes associated with regions that are less accessible in the absence of *Mef2c* also revealed B-cell differentiation and V(D)J recombination among the top enriched terms (Fig. 7b). In comparison, bone marrow myeloid cells had fewer regions that showed differences (ratio≥4, *P* value≤0.01) in accessibility on *Mef2c* loss, as only 346 sites were more accessible in control and 73 in *Mef2c*-deficient myeloid cells. The sites enriched in control myeloid cells were associated with genes encoding phagocytosis and chromatin remodelling, and were distinct of those regulated by MEF2C in pre-B cells. (Supplementary Fig. 8a, Supplementary Data 5) These data suggest that MEF2C enhances the expression of DNA repair and V(D)J factors in B-lymphoid cells in part through its effects on chromatin accessibility at their regulatory sites.

Analysis of the ATAC-seq in MEF2C direct target genes (based on human B-lymphoblast and mouse B-cell progenitor BMiFLT3 ChIP-seq data) that are critical for B-cell differentiation (*Tcf3*) and DNA repair (*Mre11a, Lig4 and Xrcc4*) identified regions that are more accessible in control than *Mef2c-*deficient pre-B cells in all these genes. A majority of them were uniquely accessible in pre-B cells as compared with myeloid cells (Fig. 7c). Correlation of MEF2C-binding sites in mouse B-cell progenitors with ATAC-seq data in pre-B cells showed that MEF2C binds to open chromatin regions (Fig. 7c). However, the sites that lost accessibility in *Mef2c*-deficient pre-B cells did not typically co-localize with MEF2C binding, suggesting that the modulation of chromatin accessibility by MEF2C is indirect (Fig. 7c). Motif analysis^44^ of regions that are more accessible in control than *Mef2c-*deficient pre-B cells showed that E-box (motif for bHLH factors such as TCF3) and EBF were among the top enriched motifs (Fig. 7d). Intersection of TCF3-binding sites in mouse pro-B cells^45^ with regions that are less accessible in *Mef2c-*deficient pre-B cells showed significant overlap. Moreover, many of the peaks less accessible in *Mef2c*-deficient cells also correlated with cell type-specific regulatory regions that are more accessible in pre-B cells compared with myeloid cells (Fig. 7e). In contrast, there was minimal overlap with TCF3-binding and MEF2C-dependent accessible regions in myeloid cells (Supplementary Fig. 8b). These data suggested that MEF2C may modulate chromatin accessibility through its direct target genes, such as TCF3.

## Discussion

DNA damage repair is vital to the genomic integrity of stem/progenitor cells. In addition to serving housekeeping functions in most lineages, DNA repair and DSB repair specifically, is critical for normal lymphoid development due to its necessity during V(D)J recombination. It has been unknown, however, if B-lymphoid cells possess lineage-specific mechanisms that link the activation of DNA repair machinery with B-cell differentiation. Moreover, it is not known if there are unique requirements for B-cell transcriptional machinery during regenerative stress, when efficient DNA repair is required to protect the rapid differentiation and proliferation of stem/progenitor cells. Our work identifies MEF2C as a transcriptional activator that ensures efficient DNA repair and V(D)J recombination in bone marrow B-cell progenitors, and becomes critical for B-cell regeneration during stress haematopoiesis.

**Figure 8:**
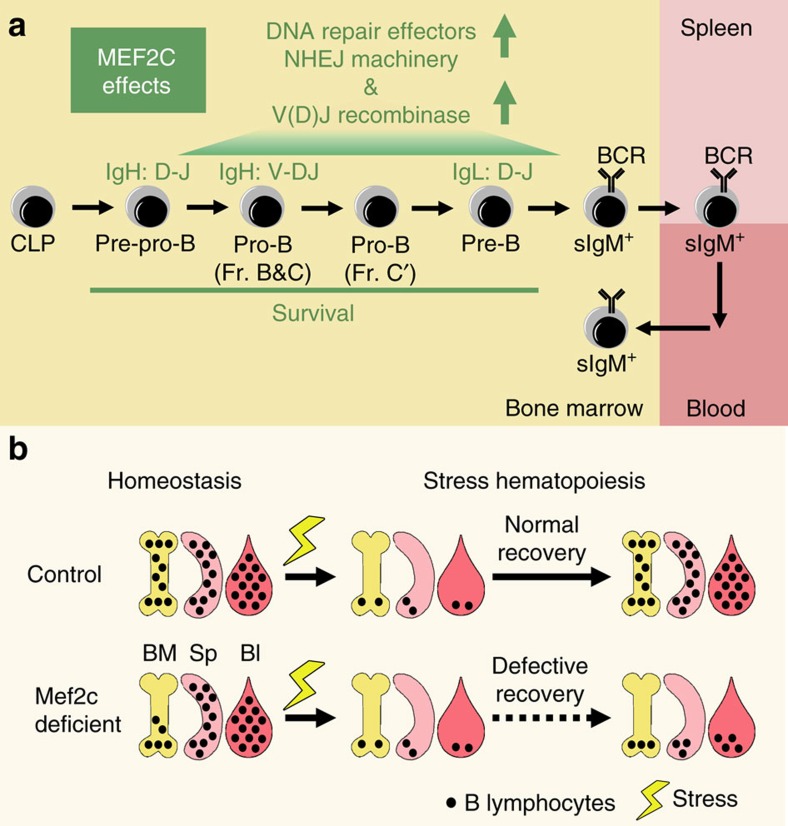
MEF2C enables B-lymphoid regeneration during stress. (**a**) MEF2C ensures efficient bone marrow B lymphopoiesis by enhancing the expression of critical V(D)J recombination initiators and DSB repair machinery thereby promoting the rearrangement of both heavy and light chains, the success of which ensures the survival of bone marrow B-cell progenitors. (**b**) While loss of MEF2C compromises bone marrow B lymphopoiesis during homeostatic conditions, this can be compensated for in the periphery to maintain a relatively intact B-cell pool in blood and spleen. However, the loss of *Mef2c* severely compromises B-lymphoid recovery after irradiation, documenting a critical function for MEF2C in regeneration of the B-lymphoid compartment during stress haematopoiesis.

We show that MEF2C enhances the transcription of genes encoding DSB repair machinery, RAG recombinases and B-lymphoid regulators in bone marrow B-cell progenitors. Thus, MEF2C enables efficient V(D)J recombination, a pre-requisite for B-lymphoid progenitor survival (Fig. 8a). Absence of *Mef2c* reduces the efficiency of both heavy and light chain rearrangement, compromising bone marrow B lymphopoiesis at multiple stages. While *Mef2c* loss leads to reduced B lymphopoiesis in homeostatic conditions, peripheral B-cell numbers are normal most likely due to homeostatic expansion of mature B cells or eventual saturation of B-cell survival niches in secondary lymphoid organs. However, during stress, such as recovery from irradiation or 5-FU treatment, the lack of *Mef2c* becomes a bottleneck for rapid bone marrow B lymphopoiesis that is required for timely replenishment of peripheral B-lymphoid compartment (Fig. 8b). Although developing T cells also undergo V(D)J recombination, our analyses show that *Mef2c* loss has essentially no effect on DNA repair in T-cell progenitors, steady state T lymphopoiesis or T-cell recovery during stress. These data document a unique requirement for MEF2C-dependent transcriptional mechanisms to secure efficient bone marrow B lymphopoiesis during stress. Such mechanisms can be important in various stress conditions such as during the recovery from bone marrow transplantation, radiation or chemotherapy. Given the high homology between MEF2 family members, it is possible that MEF2D and/or another MEF2 family member expressed in T-cells^46^ functions in developing thymocytes in a manner analogous to MEF2C in B-cell progenitors.

Quantification of B-cell progenitors shows that the B-lymphoid defects in *Mef2c*-deficient mice have similar features as observed in aged mice. This includes loss of bone marrow B-cell progenitors, especially pre-B cells, without immediate effects on peripheral B-cell compartments^11^,^12^. Although the effects of ageing in B-lymphoid cells are most notable at pre-B-cell stage, defects are already observed at CLP level. Notably, *Mef2c* deficiency also compromises the recovery CLPs during stress. Similar to *Mef2c* deficiency, ageing of the B-lymphoid compartment is associated with increased B-cell progenitor death^16^ and defective BCR rearrangement^14^. Such defects have been linked to reduced *Tcf3(E2a)* and *Rag* expression and defective DNA repair in the elderly^47^,^48^,^49^, phenomena that we also observe in *Mef2c*-deficient mice. It is intriguing that the loss of a single gene, *Mef2c*, leads to similar phenotypes in B-cell progenitors as observed during ageing. Future studies will be needed to determine at both molecular and cellular levels the degree to which *Mef2c* deficiency models physiological B-lymphoid ageing.

MEF2C ChIP-seq analysis in human B-lymphoblasts and mouse B-cell progenitors documents MEF2C binding at enhancers and/or promoters of critical B-cell transcription factors and genes essential for V(D)J recombination and DNA repair, suggesting a direct regulatory function for MEF2C. ATAC-seq data in mouse pre-B cells shows that *Mef2c* loss results in reduced chromatin accessibility also at these critical genes. Many of the regulatory sites with reduced chromatin accessibility in *Mef2c*-deficient cells overlap with TCF3-binding sites^45^, whereas the overlap with MEF2C-binding sites is marginal. Our analysis thus identify two complementary mechanisms of how MEF2C may promote these transcriptional programs: (1) MEF2C directly binds to and promotes the transcription of its target genes and (2) MEF2C indirectly regulates the chromatin accessibility of B-cell and DNA repair genes through factors such as TCF3 that it also activates. Further studies will be required to mechanistically define how MEF2C and TCF3 co-operate in regulating chromatin accessibility and gene induction. Altogether, our data suggest that MEF2C is not a *bona fide* lineage-specification factor whose absence leads to an absolute block in gene activation or lineage differentiation. Rather, MEF2C functions in ‘volume-control' to enable efficient upregulation of B-cell transcriptional programs, including factors required for V(D)J recombination and DNA repair.

*Mef2c* is also known as a cooperating oncogene in leukaemia. *Mef2c* is abnormally induced in leukaemic GMPs in myeloid leukaemia and knockdown of *Mef2c* attenuated the proliferative potential of these cells^50^. In MLL-ENL, loss of *Mef2c* in leukaemic cells reduced their homing and invasive capacities^51^. Moreover, MEF2C is emerging as an important oncogene in T-ALL^52^,^53^,^54^,^55^. It is unclear how ectopic induction of *Mef2c* promotes leukaemia progression, and whether it regulates DNA repair that could lead to survival advantage and/or therapy resistance. Our finding that MEF2C acts as an amplifier of cell type-specific transcriptional programs in B-lymphoid cells is consistent with the data that MEF2C is not an oncogene alone, but potentiates the leukemogenic effect of the oncogene it co-operates with^56^. Moreover, as MEF2C functions in normal development of various other tissues (for example, muscle, heart, vasculature and neural progenitors)^23^,^57^,^58^,^59^,^60^, it is important to define if MEF2C functions similarly as in B-lymphoid cells to augment cell type-specific transcriptional programmes in these stem/progenitor cells.

## Methods

### Mice

Vav-Cre mice were bred with *Mef2c*^fl/fl^ mice to generate Vav-Cre *Mef2c*^fl/fl^ mice. *Mef2c*^fl/fl^, *Mef2c*^fl/+^ or Vav-Cre *Mef2c*^fl/+^ mice of the same age were used as controls. Genotyping PCR primers are: Cre-F 5′-CGTATAGCCGAAATTGCCAG-3′ and Cre-R 5′-CAAAACAGGTAGTTATTCGG-3′ (Cre band, 200 bp), Mef2c-3′arm 5′-CTACTTGTCCCAAGAAAGGACAGGAAATGCAAAAATGAGGCA-3′ and Mef2c-KO region 5′-GTGATGACCCATATGGGATCTAGAAATCAAGGTCCAGGGTCAG-3′ (wild-type band, 586 bp; flox band, 838 bp) and Mef2c-3′arm (listed in this section) with Mef2c-3′neo 5′-GTGGGCTCTATGGCTTCTGAGGCGGAAAG-3′ (mutant band, 253 bp). All mouse experiments were approved and maintained in accordance with the UCLA Animal Research Committee (ARC)/Institutional Animal Care and Use Committee (IACUC).

### Flow cytometric analysis and isolation of B-cell progenitors

Haematopoietic cells were analysed using antibodies listed in Supplementary Table 1. Dead cells were excluded with 7-amino-actinomycin D and cell populations were analysed using a LSR II or Fortessa flow cytometer. Cell sorting was performed using a FACS Aria cell sorter. Data were analysed with FlowJo software version 9.2. Fractionation of bone marrow B-lymphoid progenitors was based on the surface expression of the following markers: pre-pro-B, B220^+^IgM^−^CD43^+^CD24^−^; pro-B, B220^+^IgM^−^CD43^+^CD24^+^; pre-B, B220^+^IgM^−^CD43^−^ and sIgM^+^, B220^+^IgM^+^. Hardy fractionation of bone marrow B-lymphoid progenitors was based on the surface expression of the following markers: Fr.A, B220^+^IgM^−^CD43^+^CD24^−^BP-1^−^; Fr.B, B220^+^IgM^−^CD43^+^CD24^+^BP-1^−^; Fr.C, B220^+^IgM^−^CD43^+^CD24^−^BP-1^+^; Fr.D, B220^+^IgM^−^CD43^−^; Fr.E, B220^+^IgM^+^IgD^−^; Fr.F, B220^+^IgM^+^IgD^+^. For the antibody target, clone, dilution and source of all flow cytometric antibodies, please see Supplementary Table 1.

### Caspase 3/7 activation measurement

Activation of caspase 3/7 was detected by CellEvent Caspase 3/7 Green Detection Reagent based on instructions from the manufacturer. Specifically, cells were first stained with antibodies for surface markers as described above. Cells were then resuspended in 5% FBS PBS to reach 1 × 10^7^ cells per ml, after which 1 μl of CellEvent Caspase 3/7 Green Detection Reagent was added. Samples were then incubated at 37 °C for 30 min, protected from light. Samples were subjected to Fortessa flow cytometer and Caspase 3/7 activity was detected through FITC channel.

### Induction of stress haematopoiesis

Two different methods were used to induce stress haematopoiesis. Sub-lethal (6 Gy) total body irradiation was performed with Co-60 pool irradiator. Ablation of the haematopoietic cells without irradiation was caused by 5-FU treatment. 5-FU (Sigma) was dissolved in sterile PBS and mice were i.v. injected with a single dose of 150 mg kg^−1^.

### Analysis of peripheral blood counts

Peripheral blood was harvested from the retro-orbital sinus into Vacutainer tubes (BD Biosciences). Samples were sent to UCLA Division of Laboratory Animal Medicine laboratory for complete blood cytometry analysis.

### Gene expression profiling

Total RNA was isolated from sorted cells using a combination of QIAshredder columns followed by the RNEasy Micro/Mini Kit (QIAGEN). Affymetrix microarray analysis was performed on independent control and *Mef2c*-deficient, sorted pro-B- and pre-B-cell samples. The R package Limma provided through the open source project Bioconductor was used to assess differential expression. To calculate absolute mRNA expression levels, the robust multiarray averaging method was used to obtain background adjusted, quantile normalized and probe level data summarized values for all probe sets. The Affymetrix Mouse Genome 430 2.0 Array GeneChip platform was used for the analysis. Official gene symbols for probe sets were obtained using the Bioconductor annotation database mouse4302.db. The mas5calls algorithm through the R package of affy was used for calculating PMA detection calls for each array sample. Probes that were absent or only marginally present in more than four replicates were excluded from analysis. Differentially expressed genes were uploaded into the DAVID interface to identify significantly over-represented functional GO biological process categories.

### Quantitative reverse transcription–PCR

Total RNA was extracted from B-cell progenitors (see above). cDNA synthesis was carried out according to the manufacturer's protocol for the Quantitect Reverse transcription kit (QIAGEN), and Q-PCR was performed using a LightCycler 480 (Roche) with LightCycler 480 SYBR Green I Master mix (Roche). Primer sequences are shown in Supplementary Table 2. Samples were normalized to *Actb*.

### Immunofluorescence microscopy (γH2AX)

IF microscopy of γH2AX was performed as described. In brief, FACS-purified cells were pipetted onto poly-lysine-coated slides, fixed with 4% PFA for 10 min at room temperature, permeabilized in 0.15% Triton X-100 for 2 min at room temperature and blocked in 10% donkey serum/PBS overnight at 4 °C. Slides were then incubated for 1-2 h at room temperature with anti-phospho-H2AX (Ser 139) (Millipore, 05-636). Slides were washed three times in PBS and incubated for 1 h at room temperature with AF488-conjugated goat anti-mouse (Life Technologies, A11029) antibody. Slides were then washed three times in PBS and mounted using ProLong Gold Antifade Reagent with DAPI (Life Technologies, P36935). Microscopy imaging was performed using Zeiss LSM 700 confocal microscope (× 100 objective) and Nikon ECLIPSE E600 microscope (× 100 objective). Cells were scored as positive (≥4 foci) or negative (0–3 foci) based on the number of foci observed by eye. All scoring was done blind and >50 cells were counted per sample.

### Alkaline comet assay

Alkaline comet assay was performed with Enzo comet SCGE assay kits according to the manufacturer's protocols. In brief, FACS-purified cells were embedded in low-melting point agarose and transferred onto comet slides. Cells were lysed and treated with freshly made alkaline solution followed by electrophoresis in alkaline electrophoresis solution. Slides were dried for at least 2 days before imaging. Nuclei were stained with SYBR Green I for 20 min. Pictures of individual cells were taken with Nikon ECLIPSE E600 microscope (× 40 objective) and analysed using the CASP software (http://casplab.com/). The tail moments of all comets analysed were used to define outliers and non-outliers based on calculated absolute deviation. Cells were defined as outliers when the absolute deviation of tail moments was ≥3 median absolute deviations.

### Quantitative analysis of Ig gene rearrangements

Genomic DNA was extracted from sorted B-cell populations (pro-B for heavy chain and pre-B for light chain) with the genomic DNA extraction kit (QIAGEN). Quantitative analysis of V_H_-J558 and V_H_-7183 heavy chain and κ and λ light chain rearrangements were performed by Q-PCR using published primers^61^. Q-PCR was performed as stated above. Samples were normalized to *Actb*. Rearrangement frequencies were calculated as 2^ΔCt^ with ΔCt=Ct_*Actb*_–Ct_*Ig*_.

### ChIP-seq analysis of ENCODE project

Alignment images were generated with the UCSC Genome Browser^62^ and identification of peaks was based on the peak calling process of the ENCODE project. Peak intersection was done with Galaxy^63^ and peaks were mapped to nearby genes using the default setting of Genomic Regions Enrichment of Annotations Tool (Great)^64^. The following publicly available ChIP-seq data sets generated with GM12878 cells from the ENCODE^42^ project were used for analyses: MEF2C GSM803420, p300 GSM935562, H3K4me1 GSM733772, H3K4me3 GSM733708, H3K27ac GSM733771, H3K9me3 GSM733664, H3K27me3 GSE50893.

### ChIP-seq analysis in BMiFLT3 mouse B-cell progenitors

Replicates of MEF2C IPs and IgG controls reads were combined and mapped to mm9 mouse genome. Duplicated reads mapping to the same position in the genome were counted only once to reduce clonal amplification effects. Each read was then extended by 150 bases. Mouse genome was split into 50-bp windows. Total read counts of input and IP samples were normalized against each other and normalized read counts were assigned to each of the 50-bp windows. Peak enrichment analysis was performed by calculating the probability of observing the IP sample counts within a window given the expected counts in the input sample window using Poisson distribution. For a window to have significant enrichment over background, the window needs to have *P* value <1.0 × 10^−3^ and both of its neighbouring windows also need to have *P* value <1.0 × 10^−3^. The ChIP-seq peak-finding algorithm was implemented in MATLAB. The following publicly available ChIP-seq data sets^30^ were used for analysis: GSM1894133, GSM1894135, GSM1894134, GSM1894136.

### ChIP-seq analysis of histone marks in mouse pre-B cells

FACS-sorted bone marrow pre-B cells (7–10-month old) were washed and crosslinked 10 min at room temperature with 1% formaldehyde in PBS plus protease inhibitor (PI). Glycine was added at a final concentration of 125 mM and tubes were further mixed for 5 min at room temperature. Following washes with PBS, ChIP reactions were performed with the Diagenode True MicroChIP X16 kit. In brief, cells were lysed in buffer tL1 plus PI on ice for 5 min. Subsequently, lysates were diluted with PBS plus PI and sonicated using a Diagenode Bioruptor with a chilled water bath. Lysates were then diluted with an equal volume of buffer tC1 plus PI and rotated at 4 °C overnight after the addition of 0.5 μg of anti-H3K27ac (Abcam), anti-H3K4me3 (Diagenode) or Rabbit IgG (Diagenode). The next day, beads that prepared following manufacturer guidelines plus immune complexes were subsequently washed with buffers tW1–tW4. ChIPed DNA was eluted with buffer tE1 for 30 min at room temperature. In parallel, input DNA equivalent to 10% of each ChIP was diluted with buffer tE1. Buffer tE2 was added to each sample and crosslinks were reversed via overnight incubation at 65 °C. The next day, DNA was purified via the DNA Clean and Concentrator-5 kit (Genesee Scientific) following the standard protocol. ChIP-Seq library preparation was carried out with the NuGen Ovation Ultralow System V2 1-16. Library amplification was performed for 15 cycles. All library preparation steps were carried out as stated in the user manual. Purified libraries were diluted and mixed at equal molar amounts. Pooled libraries were multiplexed and sequenced (50 bp, single end) on an Illumina HiSeq 2000. Demultiplexing of pooled samples was performed with in-house shell scripts. Reads were then mapped to the mouse mm9 reference genome using bowtie^65^ retaining only uniquely mapping reads with two mismatches. Peaks were identified with MACS14 (ref. 66) using respective input as a reference.

### ATAC-seq analysis

ATAC-seq was performed in 2–3 biological replicates using 50,000 FACS-sorted myeloid cells (Mac1^+^Gr1^+^) and pre-B cells (B220^+^sIgM^−^CD43^−^) from the bone marrow of control and Vav-Cre *Mef2c*^fl/fl^ mice. Briefly, after nuclear extraction, transposition reaction (Nextera DNA Library Prep Kit, Illumina) was carried out at 37 °C for 30 min. After DNA purification, 12 cycles of PCR amplification was carried out using NEBNext High-Fidelity 2 × PCR Master Mix (New England Labs). Purified libraries were subsequently subjected to paired end sequencing using HIseq 2000 (Illumina) to obtain 50-bp-long reads. Demultiplexing was carried out using in-house Unix shell script followed by mapping to mouse genome (mm9) using Bowtie^65^. Peaks were identified with MACS^66^, and MAnorm analysis^43^ was used to identify differentially accessible regions between control myeloid and pre-B cells, as well as control and *Mef2c-*deficient pre-B cells or myeloid cells. Peaks that had at least four times more reads in one condition and had *P* value≤0.01 were considered differentially accessible. Centdist analysis^44^ was used to identify transcription factor-binding motifs enriched within different subsets of differentially accessible regions. GREAT program^5^ was used to associate identified regions with genes (using default settings) and to perform GO-enrichment analysis.

### Statistics

For all the experiments other than genome-wide sequencings, student's unpaired two-tailed *t*-test was used for statistical analysis. Differences with *P* values≤0.05 were considered significant.

### Data availability

Microarray and sequencing data that support the findings of this study have been deposited in GEO with the primary accession codes GSE63996, GSE63997, GSE81646 and GSE81508. The authors declare that all other data supporting the findings of this study are available within the article and its supplementary information files.

## Additional information

**How to cite this article:** Wang, W. *et al.* MEF2C protects bone marrow B-lymphoid progenitors during stress haematopoiesis. *Nat. Commun.* 7:12376 doi: 10.1038/ncomms12376 (2016).

## Supplementary Material

Supplementary Information Supplementary Figures 1-8 and Supplementary Tables 1-2

Supplementary Data 1 Gene expression analysis of control and Vav-Cre *Mef2c^fl/fl^* pre-B cells

Supplementary Data 2 Gene expression analysis of control and Vav-Cre *Mef2c^fl/fl^* pro-B cells

Supplementary Data 3 MAnorm analysis of ATAC-seq peaks in control pre-B and myeloid cells

Supplementary Data 4 MAnorm analysis of ATAC-seq peaks in control pre-B and *Mef2c* deficient pre-B cells

Supplementary Data 5 MAnorm analysis of ATAC-seq peaks in control myeloid and *Mef2c* deficient myeloid cells
