# Supplementary material for: MEF2C protects bone marrow B-lymphoid progenitors during stress haematopoiesis
Source: Nat Commun. 2016 Aug 10;7:12376. doi: 10.1038/ncomms12376 (PMC4987520; doi:10.1038/ncomms12376)
Supplement: Supplementary Information — Supplementary Figures 1-8 and Supplementary Tables 1-2 [file ncomms12376-s1.pdf]

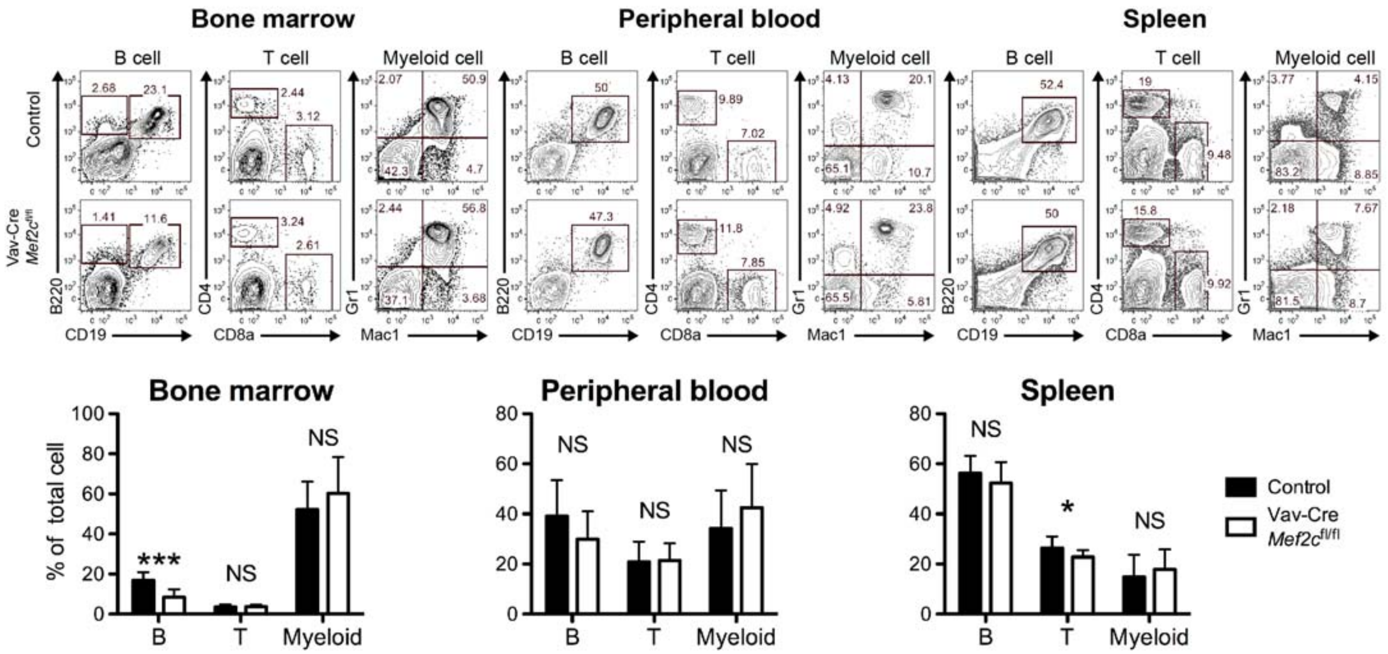

Supplementary Figure 1. Loss of *Mef2c* affects the frequency of bone marrow B-lymphoid progenitors. Deletion of *Mef2c* in haematopoietic cells in Vav-Cre *Mef2c*$^{fl/fl}$ mice resulted in specific reduction of bone marrow B cells, while the cellularity of blood and spleen B cells was unaffected (n≥7 mice). Representative flow cytometric analysis and quantification of haematopoietic lineages in bone marrow peripheral blood and spleen are shown. All mice were analyzed at 7-10 months of age and both male and female mice were included. Data shown are the mean ± SD of three or more independent experiments. NS not significant, * P<0.05, ** P<0.01 and *** P<0.001, unpaired t-test.

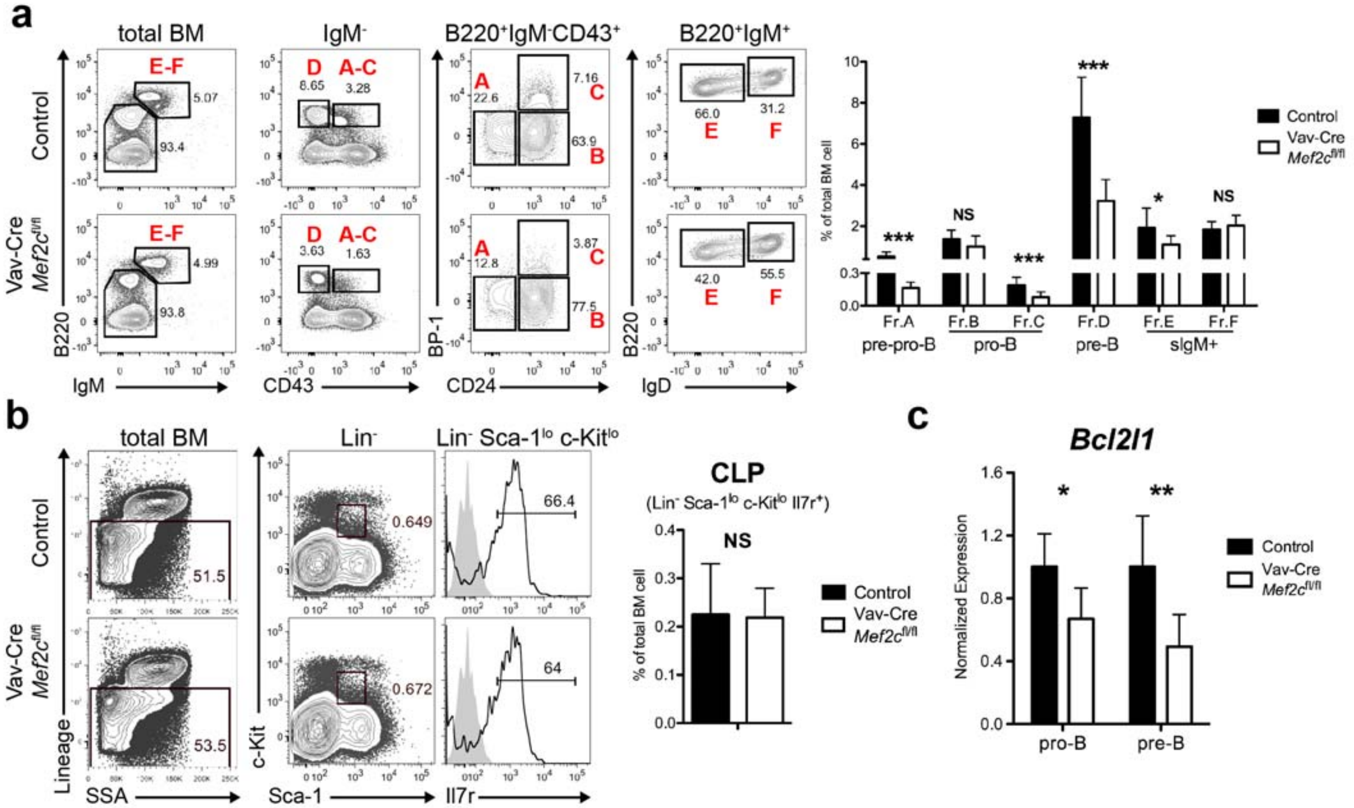

Supplementary Figure 2. Alternative staining confirms reduced B-cell progenitors in *Mef2c* deficient mice. (a) Representative flow cytometric analysis and quantification of bone marrow B-lymphoid progenitors using Hardy fractionation confirms reduction of all bone marrow B-cell progenitor subsets (n≥11). **(b)** Representative flow cytometric analysis and quantification of bone marrow common lymphoid progenitors, defined as Lin^{-}IL-7R^{+}Sca-1^{lo}c-Kit^{lo} (n≥14), shows no difference in the absence of *Mef2c*. **(c)** Q-PCR analysis of pro-survival gene, *Bcl2l1*, showed that loss of *Mef2c* compromised the expression of *Bcl2l1* in B-cell progenitors (n≥5). All mice were analyzed at 7-10 months of age and both male and female mice were included. Data shown are the mean ± SD of two or more independent experiments. NS not significant, * P<0.05, ** P<0.01 and *** P<0.001, unpaired t-test.

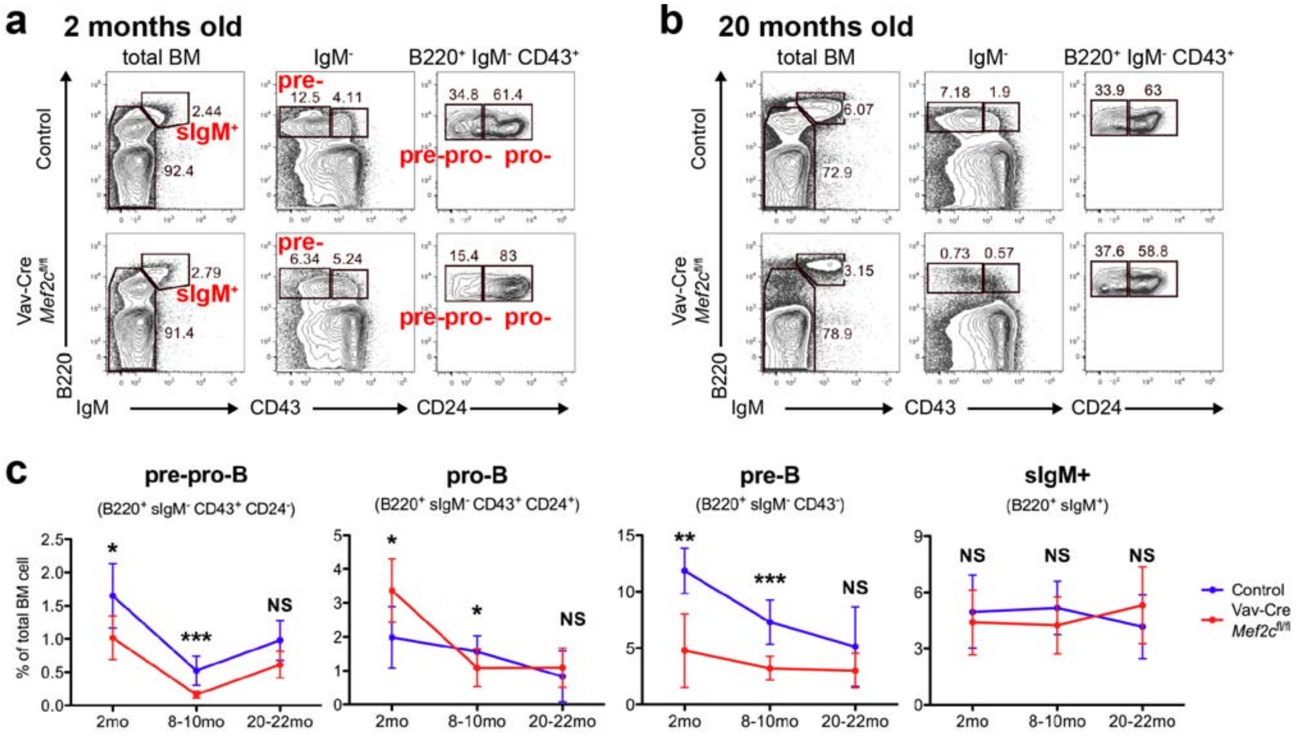

Supplementary Figure 3. *Mef2c* deficiency results in decline of B-cell progenitors that resembles ageing. (a,b) Representative flow cytometric analysis of bone marrow B-lymphoid progenitors from young (2 months) and old (20 months) mice. **(c)** Quantification of bone marrow B-lymphoid progenitors in *Mef2c* deficient and control mice of different ages shows that young/middle aged *Mef2c* deficient mice show similar reduction of bone marrow B-cell progenitors as observed in control mice during ageing (n≥6). Both male and female mice were included. Data shown are the mean ± SD of three or more independent experiments. NS not significant, * P<0.05, ** P<0.01 and *** P<0.001, unpaired t-test.

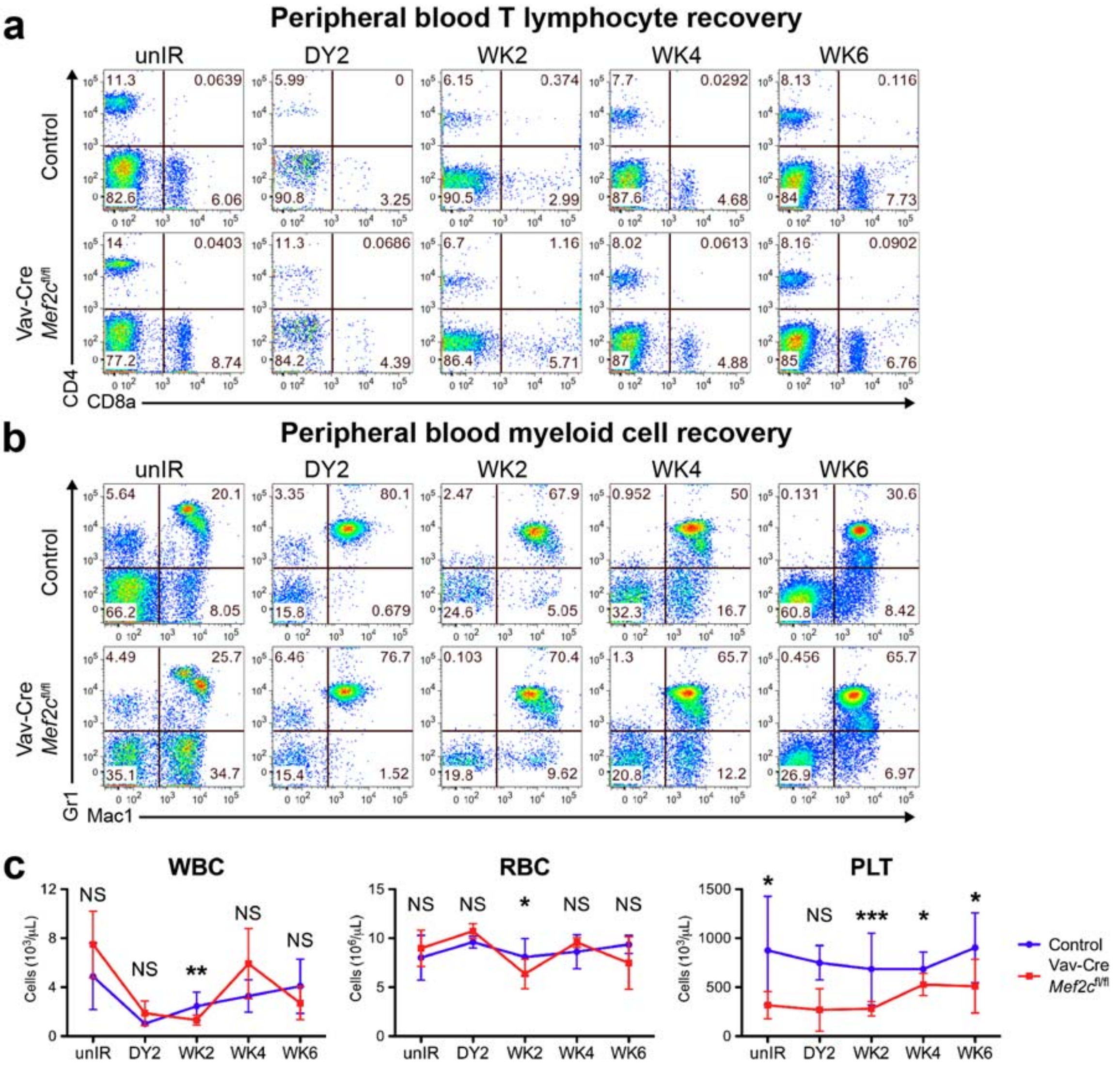

Supplementary Figure 4. Recovery of most blood lineages is not affected in the absence of *Mef2c*. (a,b) Representative flow cytometric analysis of peripheral blood T-lymphoid and myeloid cells shows comparable recovery in *Mef2c* deficient mice upon 6Gy sub-lethal irradiation as controls. **(c)** Quantification of peripheral blood white blood cell (WBC), red blood cell (RBC) and platelet (PLT) counts before and after 6Gy irradiation shows a significant defect in *Mef2c* deficient mice only in platelets, regardless of the presence of irradiation. All mice were analyzed at 7-11 months of age and both male and female mice were included. Day 2: n=4, data shown are the mean ± SD of two independent experiments; other time points: n≥5, data shown are the mean ± SD of three or more independent experiments. NS not significant, * $P<0.05$, ** $P<0.01$ and *** $P<0.001$, unpaired t-test.

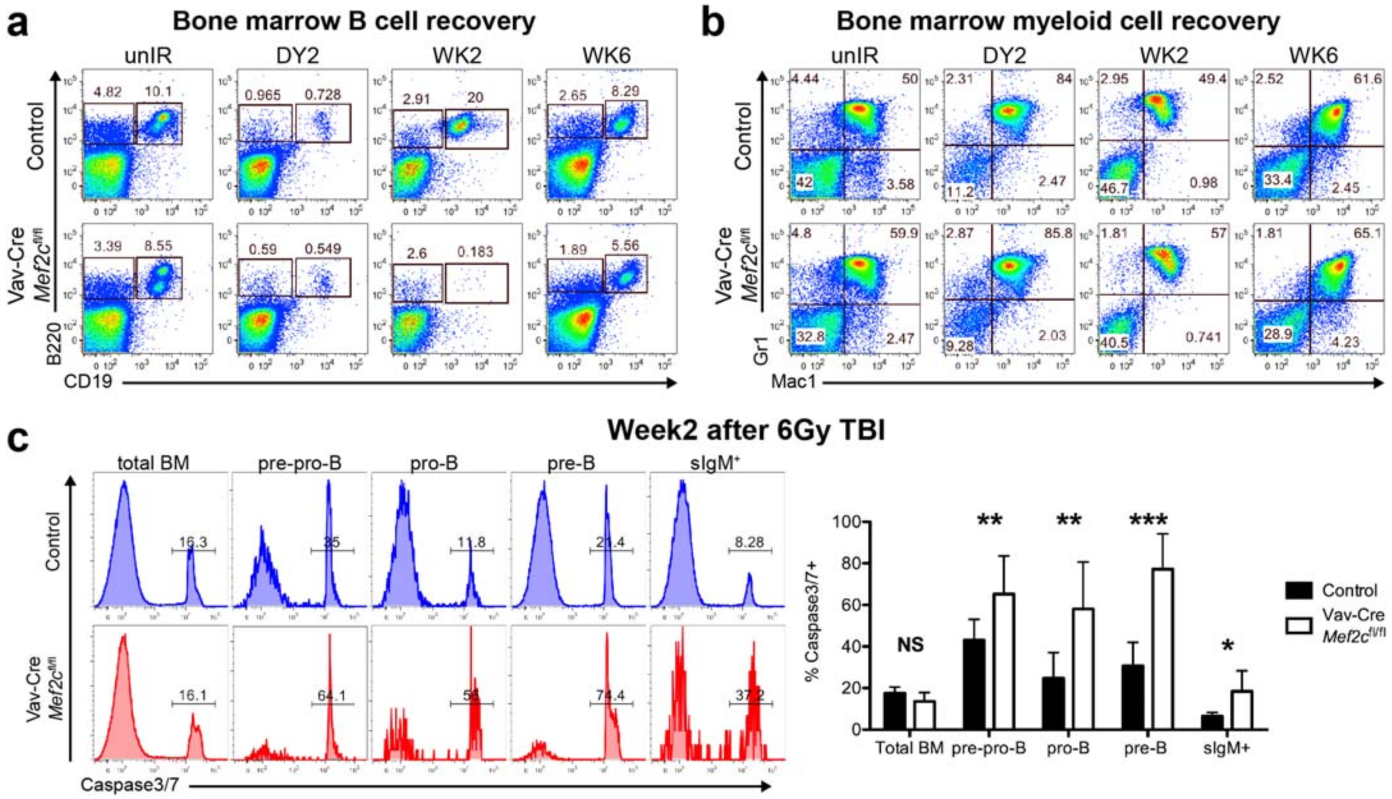

Supplementary Figure 5. Loss of *Mef2c* affects the recovery of bone marrow B cells but not myeloid cells. (a,b) Representative flow cytometric analysis of bone marrow B-lymphoid and myeloid cells shows that loss of *Mef2c* specifically compromises the recovery of bone marrow B-lymphoid cells while the recovery myeloid cells is unaffected. **(c)** Representative flow cytometric analysis and quantification of caspase3/7 activation in bone marrow B-lymphoid populations at week 2 after 6Gy irradiation (n≥5) shows compromised cell survival in *Mef2c* deficient B-lymphoid populations, but not total bone marrow cells. All mice were analyzed at 7-11 months of age and both male and female mice were included. Data shown are the mean ± SD of two or more independent experiments. NS not significant, * P<0.05, ** P<0.01 and *** P<0.001, unpaired t-test.

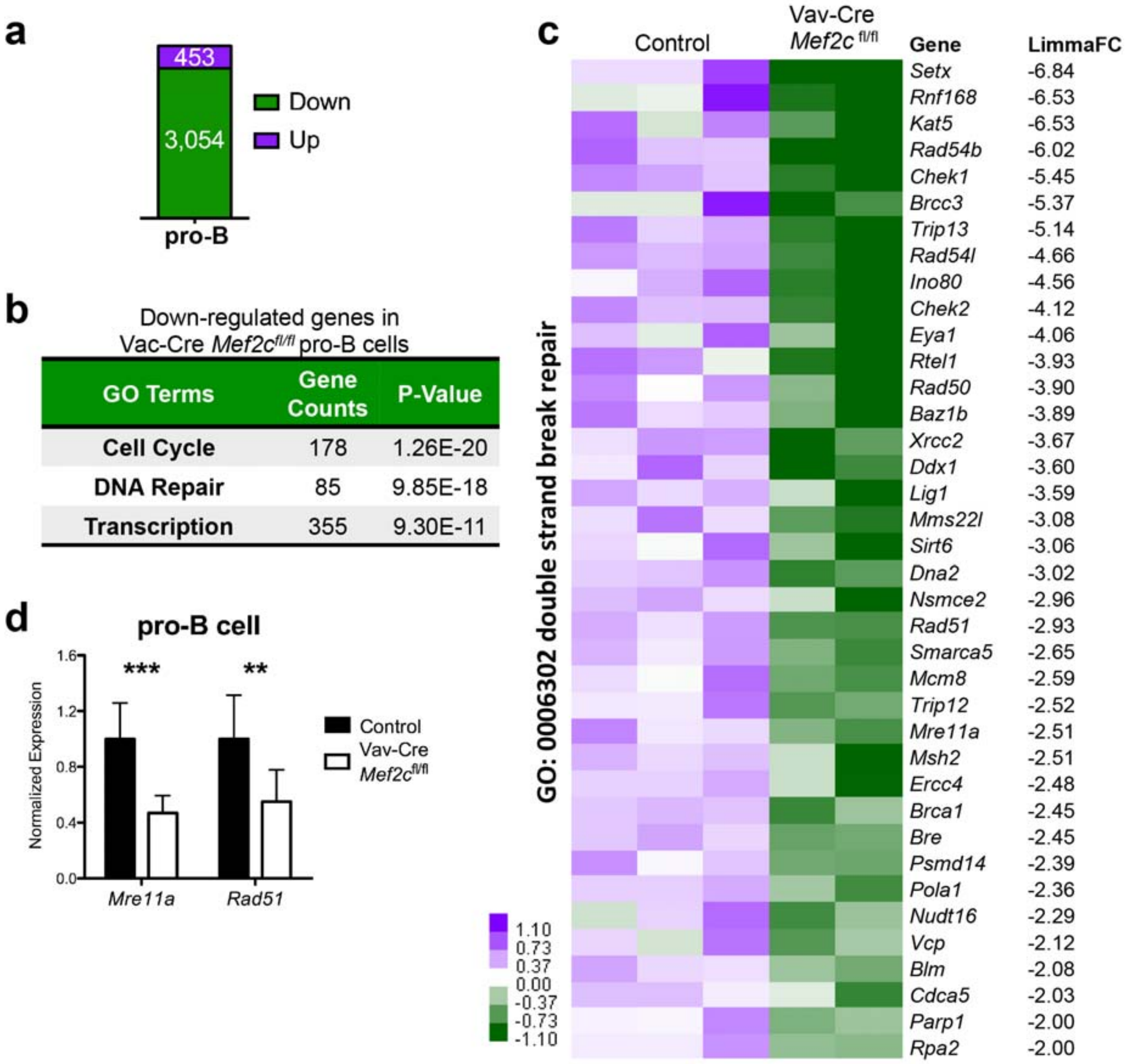

Supplementary Figure 6. MEF2C regulates DNA repair pathways also in bone marrow pro-B cells. (a) Microarray analysis of bone marrow pro-B cells from control and Vav-Cre Mef2c$^{fl/fl}$ mice (9 months old) identified 3,054 significantly (|FC|≥2 and p≤0.05) down-regulated and 453 up-regulated genes in the absence of *Mef2c* (n≥2). **(b)** Cell cycle, DNA repair and transcription were among the most significant GO categories down-regulated also in *Mef2c* deficient pro-B cells. **(c)** DNA double strand break repair factors that are significantly down-regulated in *Mef2c* deficient pro-B cells are shown. **(d)** Q-PCR of key genes encoding DNA repair machinery validated defective expression in *Mef2c* deficient pro-B cells. n≥5 mice, both male and female mice were included, and data shown are the mean ± SD of two or more independent experiments. ** P<0.01 and *** P<0.001, unpaired t-test.

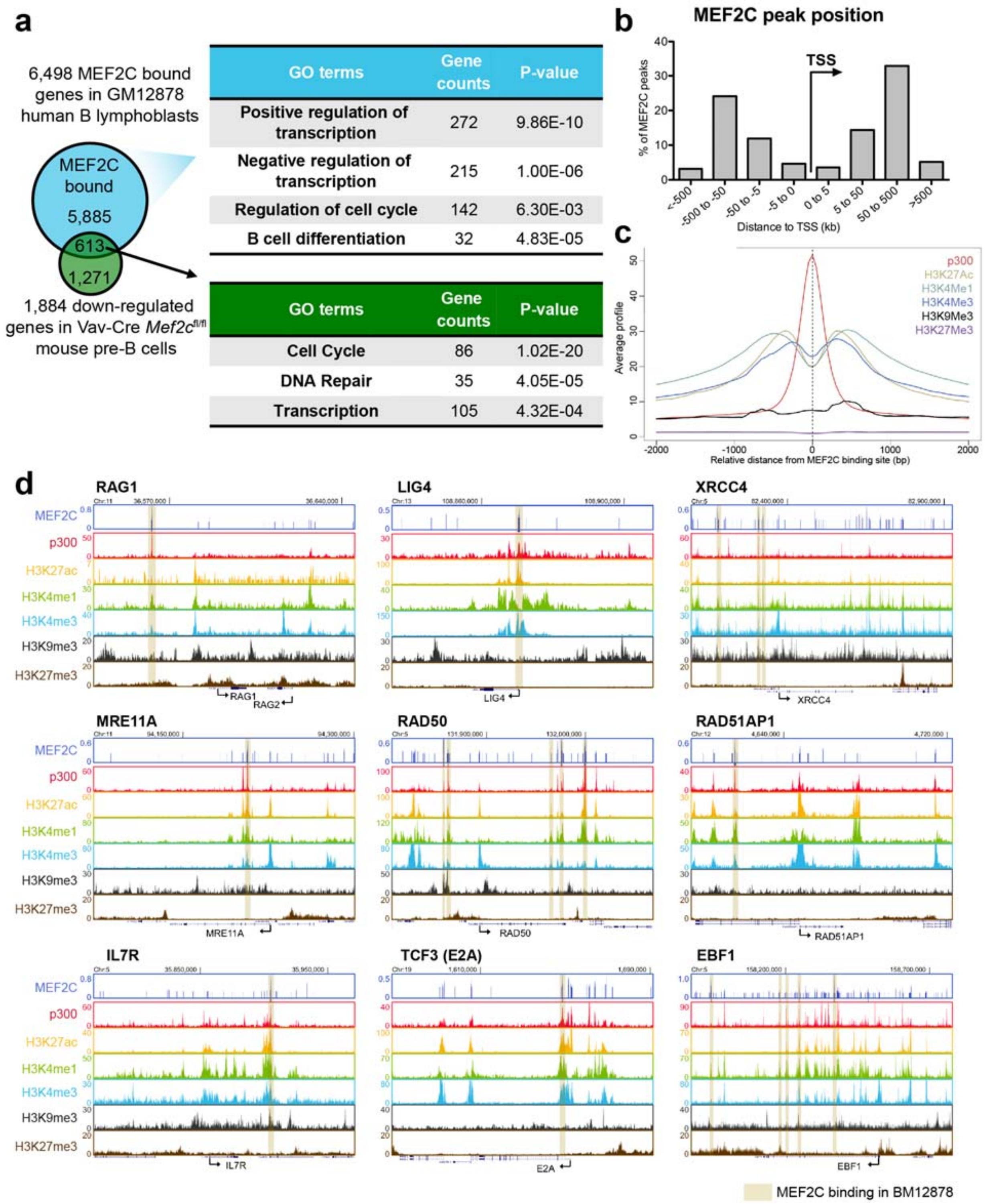

Supplementary Figure 7. MEF2C binds to active DNA repair and V(D)J genes in human B-lymphoid cells.(a) Intersection of MEF2C bound genes in human B-lymphoblasts and down-regulated genes in *Mef2c* deficient mouse bone marrow pre-B cells identified DNA repair regulators

as candidate direct targets of MEF2C in pre-B cells. **(b)** GREAT analysis showed that MEF2C binding sites are located both around TSS and at distant regulatory elements. **(c)** Distribution of co-activator p300 and epigenetic marks around MEF2C binding sites shows correlation of MEF2C peaks with both active enhancer and promoter marks (H3K4me1, H3K4me3, H3K27ac) but not repressive epigenetic marks (H3K9me3, H3K27me3). **(d)** Genome browser tracks show MEF2C and p300 binding and enrichment of active histone marks at genes encoding for DNA repair, V(D)J recombination and B-lymphoid factors. MEF2C peaks defined by the ENCODE dataset are highlighted in brown.

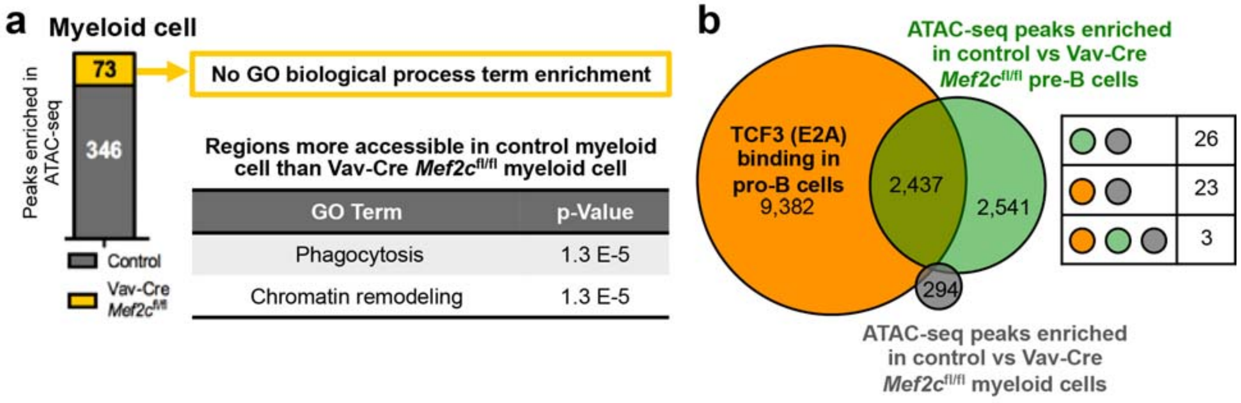

Supplementary Figure 8. MEF2C rarely promotes chromatin accessibility in myeloid cells at distinct sites. (a) MAnorm analysis of ATAC-sequencing (ATAC-seq) data in control myeloid and *Mef2c* deficient myeloid (Mac1$^+$Gr1$^+$) cells revealed that *Mef2c* loss only significantly (ratio≥4, p-Value≤0.01) affected accessibility of a small number of regulatory sites in bone marrow myeloid cells. **(b)** Venn diagram showing minimal overlaps between regions that are more accessible in control myeloid cells vs. *Mef2c* deficient myeloid cells, with regions that are more accessible in control pre-B vs. *Mef2c* deficient pre-B cells, or TCF3 bound regions in pro-B cells.

#
#
#
#
#
#
#
#
#
#
#
#
#
#
#
#
#
#
#
#
#
#

Supplementary Table 1. List of antibodies used in the flow cytometric analysis in this paper.

Antibody	Flour	Clone	Dilution	Company
B220	APC	RA3-6B2	1:200	BD Bioscience
B220	APC Cy7	RA3-6B2	1:200	BD Bioscience
B220	PerCP Cy5.5	RA3-6B2	1:200	eBioscience
B220	PE	RA3-6B2	1:200	BD Bioscience
CD19	PE	1D3	1:200	BD Bioscience
CD127 (Il7rα)	PECy7	A7R34	1:50	eBioscience
cKit	APC	2B8	1:200	eBioscience
Sca1	PECy7	D7	1:200	eBioscience
AA4.1	PE	AA4.1	1:200	BD Bioscience
CD135 (Flt3)	Biotin	A2F10	1:200	eBioscience
Gr1	APC	RB6-8C5	1:200	BD Bioscience
Mac1	PE	M1/70	1:200	BD Bioscience
CD4	APC	RM4-5	1:200	BD Bioscience
CD8a	PE	53-6.7	1:200	BD Bioscience
IgD	FITC	11-26C.2A	1:200	BD Bioscience
IgM	PE	R6-60.2	1:200	BD Bioscience
IgM	FITC	II/41	1:200	BD Bioscience
CD43	FITC	S7	1:200	BD Bioscience
CD43	Biotin	S7	1:200	BD Bioscience
Streptavidin	APC	--	1:300	Invitrogen
CD24	PE Cy7	M1/69	1:500	BD Bioscience
BP-1	Biotin	6C3	1:200	eBioscience
Ig-κ	FITC	187.1	1:200	BD Bioscience
DAPI	--	--	1:100	AnaSpec
7AAD	--	--	1:200	BD Bioscience
B220	PECy5	RA3-6B2	1:200	BD Bioscience
Mac1	PECy5	M1/70	1:200	eBioscience
Gr1	PECy5	RB6-8C5	1:200	eBioscience
CD3	PECy5	145-2C11	1:200	eBioscience
Ter119	PECy5	TER-119	1:200	eBioscience

#

Supplementary Table 2. List of primers used in the Q-PCR analysis in this paper.

Gene	Forward Primer	Reverse Primer
Actb	CATCGTGGGCCGCTCTAG	GTAACAATGCCATGTTCAAT
Bcl2l1	GGTGAGTCGGATTGCAAG	GTTCCCGTAGAGATCCAC
Mre11a	GCTCTCAGAGAGGCCGAG	CCACCTCAATGGTCTCTG
Xrcc4	CCATTCAGCCTGGACTGC	CAGTGCCTTTCTCAGCTC
Xrcc6	GATTGGGCGAGAGACAAG	GACTCCCACTCTGACATG
Lig4	CTAGCTACCTGGGACCTG	GCGACAACAAATCCTCCG
Rad51	CGAACTGGGAAGACACAG	GTGTCAATGTACATGGCC
Il7r	CTTCAAAGGCTTCTGGAGC	CTCAGAATGGTGACACTTG
Tcf3	CCAAACTGCTCATCCTGC	CCGCTTCAAGCAGGCTGC
Ebf1	CCTCAATGGCTCAGCTGC	TGAGACCATGTTGGCTG

#
